# The development of a new crop growth model SwitchFor for yield mapping of switchgrass

**DOI:** 10.1111/gcbb.12998

**Published:** 2022-10-03

**Authors:** Yanmei Liu, Astley Hastings, Shaolin Chen, Andre Faaij

**Affiliations:** ^1^ Integrated Research on Energy, Environment and Soc—Energy and Sustainability Research Institute Groningen University of Groningen Groningen The Netherlands; ^2^ Institute of Biological and Environmental Science University of Aberdeen Aberdeen UK; ^3^ Biomass Energy Center for Arid and Semi‐Arid Lands Northwest A&F University Yangling P.R. China; ^4^ TNO, Energy Transition Utrecht The Netherlands; ^5^ Copernicus Institute for Sustainable Development Utrecht University Utrecht The Netherlands

**Keywords:** bio‐energy, biomass production, genotype‐by‐environment interaction, genotype‐specific plant growth model, marginal land, SwitchFor model

## Abstract

Switchgrass is a promising energy crop has the potential to mitigate global warming and energy security, improve local ecology and generate profit. Its quantitative traits, such as biomass productivity and environmental adaptability, are determined by genotype‐by‐environment interaction (GEI) or response of genotypes grown across different target environments. To simulate the yield of switchgrass outside its original habitat, a genotype‐specific growth model, SwitchFor that captures GEI was developed by parameterising the MiscanFor model. Input parameters were used to describe genotype‐specific characteristics under different soil and climate conditions, which enables the model to predict the yield in a wide range of environmental and climate conditions. The model was validated using global field trail data and applied to estimate the switchgrass yield potentials on the marginal land of the Loess Plateau in China. The results suggest that upland and lowland switchgrass have significant differences in the spatial distribution of the adaptation zone and site‐specific biomass yield. The area of the adaption zone of upland switchgrass was 4.5 times of the lowland ecotype's. The yield difference between upland and lowland ecotypes ranges from 0 to 34 Mg ha^−1^. The weighted average yield of the lowland ecotype (20 Mg ha^−1^) is significantly higher than the upland type (5 Mg ha^−1^). The optimal yield map, generated by comparing the yield of upland and lowland ecotypes based on 1 km^2^ grid locations, illustrates that the total yield potential of the optimal switchgrass is 61.6–106.4 Tg on the marginal land of the Loess Plateau, which is approximately twice that of the individual ecotypes. Compared with the existing models, the accuracy of the yield prediction of switchgrass is significantly improved by using the SwitchFor model. This spatially explicit and cultivar‐specific model provides valuable information on land management and crop breeding and a robust and extendable framework for yield mapping of other cultivars.

## Graphical abstract

A new cultivar‐specific switchgrass plant growth model, SwitchFor model, was developed and validated to give an accurate estimation of the switchgrass yield in a wide environment. The SwitchFor model was applied to the Loess Plateau region, which has about 12.8–20.8 Mha marginal land to estimate the yield potential. It was estimated that the Loess Plateau has a potential to produce up to 62–106 Tg switchgrass by considering the upland and lowland ecotype.

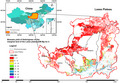

## 1 INTRODUCTION

Vigorously developing renewable energy has become a major strategic direction and concerted action for global energy transition and climate change. In China's recently released “The 14th Five‐Year Plan for Renewable energy Development,” the target for total consumption of renewable energy is to reach about 1 billion tons of standard coal, and renewable energy accounts for more than 50% of the increase in primary energy consumption in 2050 (Stern & Xie, 2022). Although no single type of renewable energy will fulfil all the targets, biofuels promise to become a significant component in the renewable energy market. Cultivation perennial grass, such as switchgrass (*Panicum virgatum* L.), on the marginal land, is characterized by a high potential in producing and providing cellulosic biomass for the production of biofuels and bio‐products (Blanco‐Canqui, 2010; Somerville et al., 2010). Moreover, it has the potential to promote biodiversity and soil carbon sequestration, mitigate climate change and environmental degradation, and contribute to the socio‐economic viability of rural (Blanco‐Canqui, 2010; Carlsson et al., 2017; Cooney et al., 2017; Lewandowski et al., 2003).

Switchgrass is a perennial C4 energy crop that displays tremendous diversity in morphology and growth habitat due to genetic diversity (Aurangzaib et al., 2018). The genotypes of switchgrass are categorized as either upland or lowland ecotypes, based on their habitat preference, morphological characteristics, and ploidy level (Brunken, 1975; Porter, 1966). The lowland ecotypes are mostly tetraploid with 2*n* = 4*x* = 36 chromosomes, whereas upland ecotypes are tetraploid and octaploid with 2*n* = 8*x* = 72 chromosomes (Hultquist et al., 1997; Lu et al., 1998). The lowland ecotypes usually have thicker stems and delayed flowering and are naturally distributed in wet and warm regions at lower latitude, whereas the upland ecotypes have thin stems and are mostly found at higher latitude in dryer and colder regions (Casler, 2005; Grabowski et al., 2004; McLaughlin & Kszos, 2005). The biomass yield of switchgrass varies greatly among genotypes and under different environmental conditions. Environmental factors such as photoperiod, temperature, and precipitation influence the expression of allelic variation and, thus, switchgrass phenotype, growth, and biomass production (Casler, 2012; Tornqvist et al., 2018). Consequently, the switchgrass biomass yield is determined by genotype‐by‐environment interaction (GEI) or the response of genotypes grown across different target environments. Therefore, to simulate the growth of switchgrass and estimate the yield outside its original region, a model should be able to capture GEI.

The Loess Plateau, China, is one of the most erosion‐prone regions in the world (Cai, 2001; Wang et al., 2006), which was estimated to have 12.8–20.8 million hectares (M ha) of marginal land available to cultivate energy crops (Liu et al., 2021). To produce the bio‐energy on the marginal land of the Loess Plateau, an accurate estimation of the switchgrass yield is a fundament for further environmental and socio‐economic impacts analysis. Liu et al. (2021) estimated the total potential biomass yield of switchgrass on the marginal land is up to 77 Tg using an improved fuzzy logical model, while the genotypes are not considered, which bring some uncertainties. Zhang et al. (2020) conducted a national scale estimation of switchgrass in China using EPIC model, whereas the results demonstrated that the Switchgrass cannot survive on the Loess Plateau, which is not consistent with the fact of successful establishment of switchgrass in the field trails experiment on the marginal land of the Loess Plateau (Zhang et al., 2020). As a consequence, the accurate estimation of the yield potential of the switchgrass which considers the genotypes on the marginal land of the Loess Plateau exists as a gap that needs to be filled, which is important to further bioenergy planning.

In this study, a new switchgrass plant growth model SwitchFor was developed to predict the biomass yield of switchgrass cultivars in a wide environment. SwitchFor is a universal genotype‐specific plant growth model developed for switchgrass, and the incorporated genotype‐specific parameters in the model processes capture the GEI of different genotypes (Clifton‐Brown et al., 2000; Hastings et al., 2009). When incorporating the identified photoperiod sensitivity, drought resistance, and frost tolerance as parameters for crop improvement, the SwitchFor model can extend the range of climatic conditions under which this crop can be grown economically. The model is developed from a well‐developed MiscanFor model, which has been applied widely (Hastings et al., 2009; Jiang et al., 2017). The MiscanFor model has been well adapted to woody biomass crops such as Poplar so that a new poplar plant growth model PopFor has been developed and successfully applied in a diverse environment (Henner et al., 2020). As a consequence, SwitchFor model developed in this study will also be an extended adoption of the MiscanFor model. In addition, the objectives of this study are to (1) develop a genotype‐specific switchgrass model, SwitchFor, which could be applied in a wide environment and (2) apply the model to predict a more accurate biomass yield of switchgrass on the marginal land of the Loess Plateau by regarding the high spatial heterogeneous climatic, soil, and topographic conditions of Loess Plateau region. The cultivar‐specific SwitchFor model developed in this study could be further adapted to other switchgrass cultivars when more switchgrass plant data are available, and applied to other research regions to investigate the switchgrass yield potential. In addition, the yield potential of switchgrass on the marginal land of the Loess Plateau predicted by SwitchFor model could provide valuable information for researchers to do further economic and environmental analysis, and for farmers, investors, and government to make decisions to develop bioenergy.

## 2 MATERIALS AND METHODS

**FIGURE 1:**
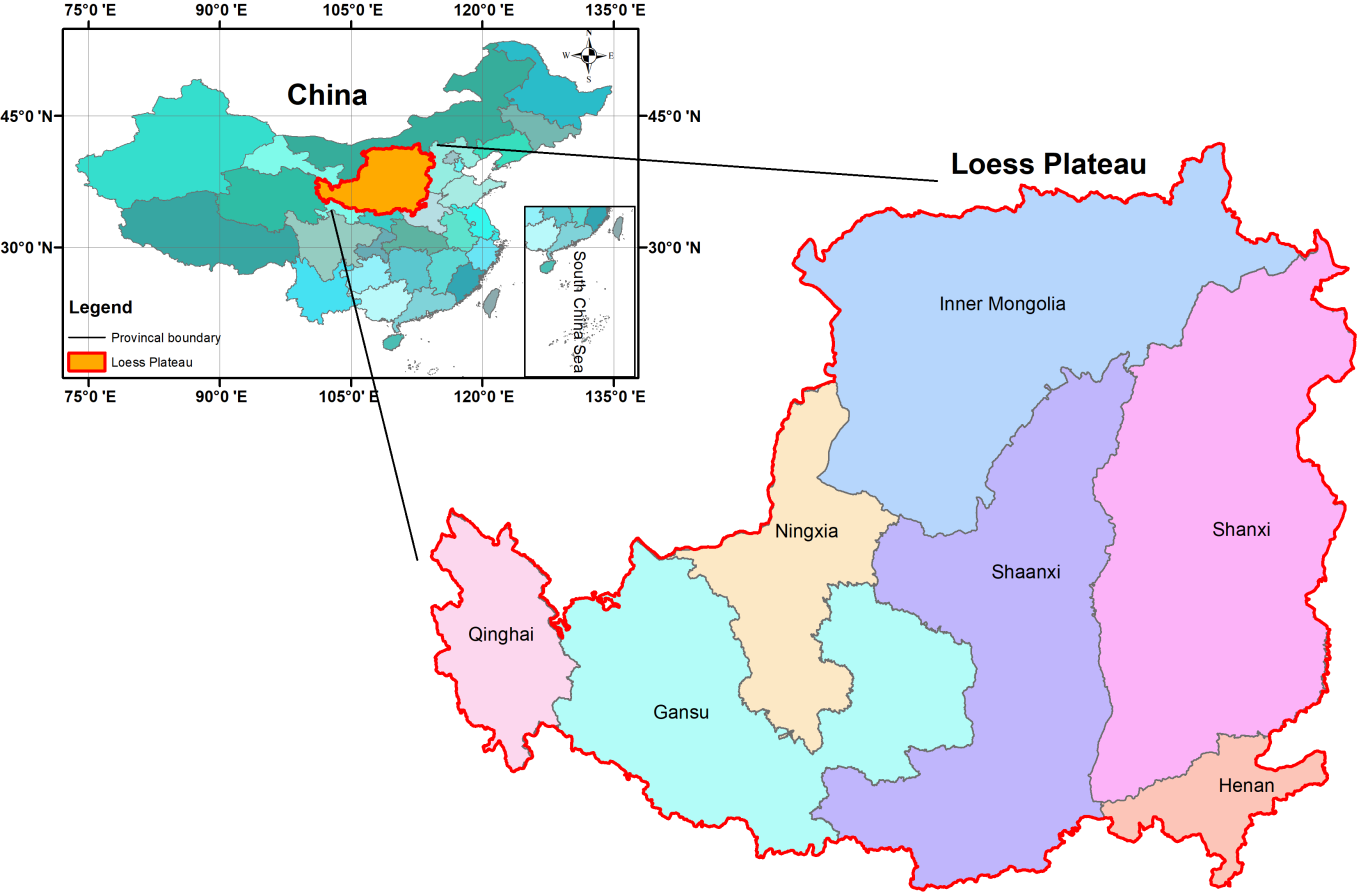
The map of Loess Plateau.

In this study, a cultivar‐specific switchgrass plant model, SwitchFor, was developed, validated, and then applied to the Loess Plateau region to estimate the yield potential on marginal land. The methodology includes the following steps. First, the key parameters and the parameterization were described in Section 2.1. The SwitchFor model was developed by parameterising the key parameters using universal data from published empirical studies on the growth and phenology of a selection of switchgrass genotypes from different lab and field trials in a variety of soil and climatic conditions. The parameters in the model were parameterized for upland and lowland switchgrass separately. Second, in Section 2.2, the model was validated based on the actual measurements from the field trials. The field trail data were collected from China, the United States, Canada, and Europe (UK, France, Germany) and thus the validation sites span a wide range of environmental condition. The detailed information of the field trails is shown in Table 3. Finally, in Section 2.3, the SwitchFor model was applied to the Loess Plateau with the spatial input data to estimate the spatial yield of the lowland and the upland switchgrass separately at a resolution of a 1 km^2^ grid cell. The overall yield potential is reflected by an optimal switchgrass yield map, which was generated by comparing the yield of upland land lowland switchgrass and extracting the higher yield in each grid cell. The yield potential on the marginal land of the Loess Plateau was generated by overlapping the regional yield map with available marginal land maps, which were identified from the previous study conducted by Liu et al. (2021). The location of the Loess Plateau is shown in Figure 1

### 2.1 SwitchFor model and key parameter description

SwitchFor is a plant growth model, which was developed based on MiscanFor (Clifton‐Brown et al., 2000; Hastings et al., 2009), which uses genotype‐specific parameters to predict yields of switchgrass in a wide range of environments. The plant growth module is driven by air temperature and incident photosynthetically active radiation (PAR). This model includes process descriptions for light interception by the canopy and the impact of temperature and water stress on radiation use efficiency (RUE), and it also includes the genotype‐specific process descriptions for plant growth phase, photo‐period sensitivity, thermal time, temperature‐dependent RUE, drought and frost kill predictions, nutrient repartition to the rhizome, and moisture content at harvest (Hastings et al., 2009). Modelling plant growth in SwitchFor requires a daily time series of maximum temperature, minimum temperature, precipitation, potential evapotranspiration (PET) and PAR, and outputs daily incremental leaf area index (LAI) and accumulated biomass in dry matter (DM) t ha^−1^ year^−1^ on a given site. In the following sections, the key processes and parameters are described.

#### 2.1.1 Soil water

**FIGURE 2:**
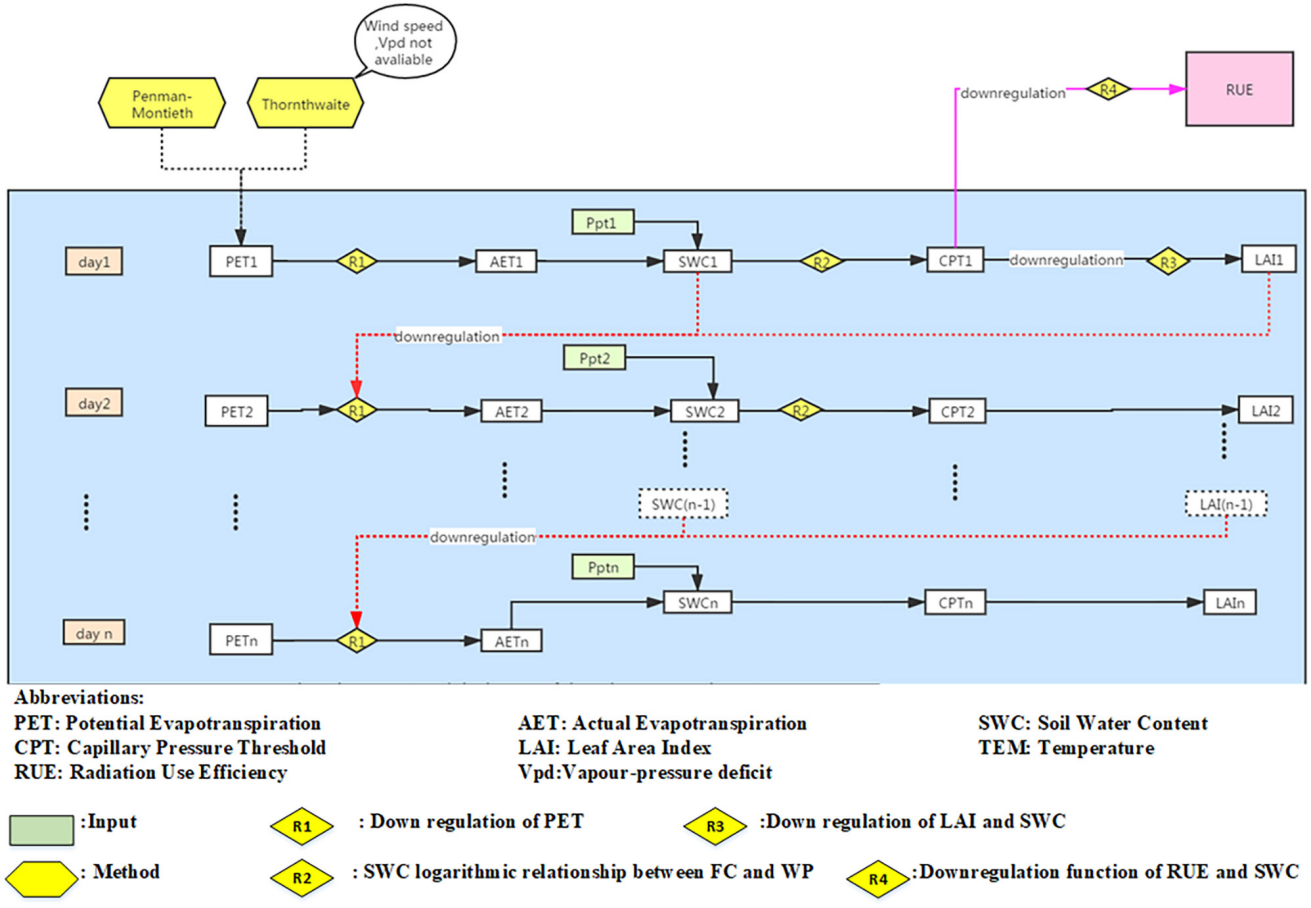
Sketch map of soil water content and the interaction with leaf area index.

Soil water stress affects both the rate of leaf expansion and photosynthesis (Luo et al., 2016; Sadras & Milroy, 1996). When the soil water content (SWC) is close to filed capacity (FC), the plant can easily extract water from the soil pore space by overcoming the soil water capillary pressure (CP). As the SWC decreases, the plant must use more energy to extract water from the increasingly small pores, experiencing water stress, until it reaches the wilt point (WP) at which the plant can no longer extract water from the soil and both growth and photosynthesis stop, resulting in the plant losing turgor and wilting. The behaviour of the plant in response to increasing water stress is genotype‐ and plant‐dependent with different thresholds for both the onset of stress and the strategy when presented with drought (Clifton‐Brown et al., 2002). In the SwitchFor model, the water stress is expressed as the soil water deficit (SWD) and its impacts on the switchgrass growth was quantified by the downregulations on the LAI and RUE. The downregulations were calculated based on the CP of the SWC by using a linear function on Ln (CP) between Ln (FC) and Ln (CPSTOP; the CP at which leaf expansion or photosynthesis ceases). The downregulation factor equalled one at FC and was zero at CPSTOP. SWC was the difference between precipitation (PpT) and actual evapotranspiration (AET). The AET was calculated as a proportion of the PET, and their relationship was determined by the interaction of the LAI and soil moisture deficit (SWD). In the model, PET was calculated using the Penman‐Montieth method or Thornthwaite method depending on the availability of the wind‐speed and relative humidity (or vapour pressure) data. The CPSTOP of leaf expansion (related to LAI) and photosynthesis (related to RUE) is species and genotype specific. FC were calculated from soil texture, geochemistry, and soil organic carbon using the Campbell method (Campbell, 1985). The WP is also species and genotype specific. Figure 2 displays a sketch map of the soil water in the model.

The soil water effects on switchgrass is based on the research of the soil moisture stress chamber experiment conducted on several upland and lowland switchgrass cultivars in greenhouse conditions (Barney et al., 2009). The drought delayed both upland and lowland switchgrass emergence from 9 to 12 days, reduced 90% of the emergence rate, and decreased the survival rate of emerged seedling by 50% when planted in −0.3 MPa compared with control (FC, 10 kPa). Once the switchgrass is well established, it shows wide moisture tolerance. Both lowland and upland ecotypes continue to produce new tillers and accumulate biomass at soil water potentials below −2 MPa, while they have 75%–80% reduction in biomass yield, tiller number, and leaf area but survived and achieved flowering below −4 MPa. Net photosynthetic rate is reduced by 50% across switchgrass ecotypes when soil water potentials were −1.5 MPa. At −11.0 MPa, all cultivars under extreme drought experienced leaf senescence and eventual necrosis with no live tissue visible at harvest, though root systems appeared intact (Barney et al., 2009). In the SwitchFor model, the CPSTOP is parameterized −2 MPa for both upland and lowland switchgrass. The impact of the soil water on the RUE and LAI was calculated using the linear downregulation factor as described before. For RUE, the linear downregulation factor was 1 when the soil water was at −10 kPa (FC) and linear downregulation factor is 0 when the soil water was at −2 MPa (WP). For LAI, the linear downregulation factor was 1 when soil water was −10 kPa (FC) and linear downregulation factor is 0 when the soil water is −1 MPa.

#### 2.1.2 Physiostat

The physiostats were defined to denote the plant growth stages in the SwitchFor model (Hastings et al., 2009). The physiostats include dormant (stage 0), shoot emergence (stage 1), leaf expansion (stage 2), leaf area maximum (leaf senescence, stage 3), plant senescence and nutrient repartition to roots and rhizome (stage 4), and above‐ground biomass drying (stage 5). The degree days (DDs) above the base temperature was used to estimate the total growing season with the constraints of the physiostat stage, and frost and drought events. The DDs also drive the physiostat clock and the phase of growth. The DDs for each stage were estimated by analysing of the best fit to the empirical observations. The parameters, which trigger each phase, include the base temperature and related DDs, actual temperature, soil moisture, and photoperiod. A detailed description of each plant growth stage and their trigger conditions is outlined in the research of Hastings et al. (2009) and is summarized in the supplement. In the SwitchFor model, all triggers, rates, and brakes calculated in the physiostat routine and the parameters are cultivar specific.

The phenological clock of switchgrass is different from *Miscanthus* × *giganteus*. Compared with miscanthus, switchgrass usually has a shorter vegetation period. The peak DM of miscanthus usually occurs in October, while the peak DM of switchgrass usually occurs in August (Heaton et al., 2008; Zeri et al., 2011). The timing and duration of the morphological development of switchgrass are cultivar dependent. The lowland switchgrass cultivar Alamo and upland switchgrass cultivar Cave‐In‐Rock (CIR) reach different stages of development on different days of the year. According to the research conducted in Texas (32°13′ N, 98°12′ W) and Virginia (37°11′ N, 80°25′ W), Alamo leaves emerge about 2 weeks earlier in spring and mature 4–6 weeks later than CIR, which results in the vegetation development duration of Alamo being 4–6 weeks longer than CIR (Sanderson & Wolf, 1995). In Texas (30°38′ N, 96°20′ W), the vegetative period of Alamo is about 120 days, while CIR is less than 60 days (Sanderson & Wolf, 1995; Van Esbroeck et al., 1997). The leaf development rate during the vegetation period of Alamo is slower than CIR, which means that it takes longer for Alamo than CIR to develop one leaf, which is one of the reasons for the longer vegetation period of Alamo. The longer vegetation period is one of the explanations behind Alamo usually reaching a higher biomass than CIR (Sanderson & Wolf, 1995; Van Esbroeck et al., 1997).

The research of Sanderson and Wolf (1995) found that moving switchgrass southward and northward affect the phenological response. When moving both Alamo and CIR southward, emergence and maturity were both occurred earlier, resulting in a longer vegetation period. This may be due to earlier emergence in spring and exposure to a shorter day length. The inverse was observed when moving plants northward (Sanderson & Wolf, 1995). Previous research has demonstrated that the morphological development of switchgrass is closely related to cumulative DDs (Sanderson & Wolf, 1995; Van Esbroeck et al., 1997).

**FIGURE 3:**
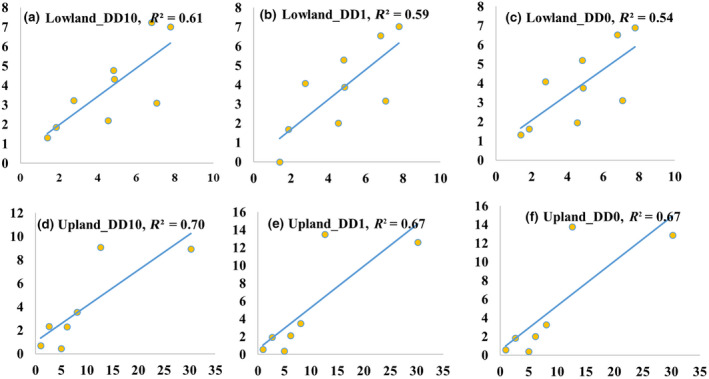
The sensitivity analysis of degree days of upland switchgrass (a–c) and lowland switchgrass (d–f) based on the field trials on the Loess Plateau.

The base temperature for DDs is different for different switchgrass growth models, and 0, 1, and 10°C is the most frequently use (Giannoulis et al., 2016; Sanderson & Moore, 1999). To parameterize the base temperature and the corresponding DDs of physiostats for Alamo and CIR in the SwitchFor model, we conducted a literature review of the DDs of the switchgrass development stages (Table S1). The day of the year (DOY) and the DDs for physiostats of Alamo and CIR vary in different locations, the average DD0, DD1, and DD10 of all the data summarized from published papers were calculated to find out the best‐fit curves with the actual measurements on the Loess Plateau. The results demonstrated that the DD10 had the best fit with the highest R2 (Figure 3). It is consistent with the previous study that the basic temperature is 10°C (Giannoulis et al., 2016; Sanderson & Moore, 1999). Therefore, a basic temperature of 10°C was used to calculate the DDs for physiostats in the SwitchFor model and the DD10 for each development stage of Alamo and CIR is shown in Table 1.

**TABLE 1:** The DD10 for physiostats of upland and lowland switchgrass.

	Stage 1	Stage 2	Stage 3	Stage 4	Stage 5
Lowland switchgrass	105	168	1174	1566	1694
Upland switchgrass	80	125	1085	1395	1488

#### 2.1.3 Leaf expansion and photosynthesis (*k*, LAI, RUE)

SwitchFor models the daily biomass DM accumulation by calculating the leaf area, solar radiation absorbed, and photosynthesis rate. The factors that affect the growth rate include temperature, leaf expansion rate modified by DDs and soil water, nitrogen availability, and RUE moderated by temperature and soil water. The values for *k*, maximum LAI, and RUE were based on the statistical analysis of the published data by an extensive review of the literature, the detailed information is shown in Table 2.

**TABLE 2:** Experimental data sets used to parameterize the key parameters for upland and lowland switchgrass in SwitchFor model.

Source	Location	Cultivars	Establishment year	RUE				LAI max		*k*
Kiniry, Johnson, Bruckerhoff, et al. (2011)	‐	‐	‐	2008	2009	2010	Mean	2008		‐
	Elsberry, US (39°9′25″ N, 90°46′55″ W)	CIR	2007	3.15	3.19	‐	3.17	2.9		‐
		Kanlow	2007	3.78	3.62	‐	3.70	4.8		‐
		Alamo	2007	5.05	3.56	‐	4.30	8.5		‐
	Gustine, US (31°53′32″ N, 98°22′29″ W)	Alamo	2007	‐	3.04	3.35	3.20	‐		‐
		Alamo	2007	‐	2.07	2.26	2.16	‐		‐
Kiniry et al. (1999)	Texas, US			1995	1996	1997	Mean	1995	1997	Mean
		Alamo	1992	4.0 ± 0.6	4.0 ± 0.7	5.3 ± 1.3	4.4	17.7	15.7	0.33
		Alamo	1993	4.4 ± 0.5	1.6 ± 0.3	5.0 ± 0.8	3.7	12.9	11.1	0.33
Madakadze, Stewart, et al. (1998)	Canada (45°28′ N, 73°45′ W)	‐	‐	1996				1996		‐
		CIR	1995	2.38				6.1		−0.54
		Pathfinder	1995	2				5.3		−0.54
		Sunburst	1995	1.96				5.1		−0.49
Heaton et al. (2008)	University of Illinois, US	‐	‐	2006				2005	2006	‐
		CIR	2005	1.2				6.5	7.2	‐
Kiniry et al. (1996) and Sanderson et al. (1996)	Tennessee, US	‐	‐	1993				1993		‐
		Alamo	1992	4.7				12		0.65
Kiniry, Johnson, Mitchell, et al. (2011)	Nebraska, US (41°13′34′ N, 96°29′18′ W)	‐	‐	‐				2008		2008
		Alamo	2002	‐				12.0 ± 5.4		−0.31
		Kanlow	2002					22.0 ± 5.6		−0.23
		CIR	2002					10.1 ± 2.9		−0.36
		Summer	2002					11.0 ± 1.0		−0.27
		Shawnee	2002					15.5 ± 5.9		−0.29
		Kanlow × Summer	2002					13.1 ± 2.0		−0.28
	Elsberry, US (39°10′09′ N, 90°47′13′ W)	‐	‐					2008		2008
		Alamo	2007	‐				8.5		−0.38
		Kanlow	2007					4.8		−0.67
		CIR	2007					2.9		−1.11
	Temple, US (31°4′ N, 97°13′ W)	‐	‐	‐				‐		1995
		Alamo	1993	‐				‐		0.34
		Alamo	1993							0.47
		Alamo	1993							0.43
		Alamo	1993							0.35
		Alamo	1993							0.34
		Alamo	1993							0.34
		Alamo	1993							0.39
		Alamo	1993							0.37
		Alamo	1993							0.44
		Alamo	1993							0.41
		Alamo	1993							0.35
		Alamo	1993							0.27
		Alamo	1993							0.36
		Alamo	1993							0.33
Trybula et al. (2015)	Purdue University, US	‐	‐	‐				8		0.5
VanLoocke et al. (2012)	University of Illinois, US (Agro‐IBIS model)	‐	‐	‐				6.5		‐
Behrman et al. (2014)	ALMANAC model	Alamo	‐	‐				12		0.33
		Blackwell	‐					6		0.33
		CIR	‐					8.8		0.36
		Kanlow	‐					6.8		0.5
Mitchell et al. (1998)	‐	Trailblazer	‐					4.9		‐
Aurangzaib et al. (2018)	Lowa, US (42°0′41″ N 93°44′34″ W)	‐	‐	‐				2012	2013	‐
		Alamo	2012	‐				5.4	4.15	‐
		Kanlow	2012					6	3.8	‐
		Cave in Rock	2012					6.64	5.06	‐
		Blackwell	2012					5.1	4.7	‐
		Trailblazer	2012					5.67	4.02	‐

Abbreviations: LAI, leaf area index; RUE, radiation use efficiency.

##### k

The statistical analysis of *k* values demonstrated that it does not display a difference between Alamo and CIR nor lowland and upland that the median value for *k* is 0.36 (Figure S1). The result is consistent with the research of Kiniry, Johnson, Mitchell, et al. (2011), which quantified a stable *k* with Beer's Law for light interception of switchgrass cultivars Alamo, Kanlow, CIR, Summer, and Shawnee based on the field trials of three locations including Texas (31°4′ N 97°13′ W), Nebraska (41°13′34′ N 96°29′18′ W), and Missouri (39°10′09′ N, 90°47′13′ W). The research of Kiniry, Johnson, Mitchell, et al. (2011) demonstrated that the *k* value of switchgrass was not related to fraction of light intercepted, time of day, or incident solar radiation. Therefore, in the SwitchFor model, the *k* value is not distinguished between upland and lowland switchgrass and 0.36 is parameterized for both switchgrass ecotypes.

##### Leaf area index

In the SwitchFor model, LAI depends on the environmental triggers that initiate growth, the rate of leaf expansion, which is proportional to cumulative DDs above a threshold, and the environmental brakes that stop leaf expansion and trigger leaf senescence. The relationship between DD_S_ and LAI was developed to indicate the leaf expansion rate modified by the DDs. During the beginning of leaf senescence, the LAI decreases from the maximum, while photosynthesis continues and DM increases until plant senescence when the peak yield is achieved. SWD moderates LAI by calculating a downregulation factor (Section 2.1.1) based on the remaining soil water CP. When DDs reach a specific value, leaf expansion stops and leaf senescence is initiated. Other triggers for leaf senescence are soil water below the WP, temperature below a critical value, first frost, and day length. These conditions also induce flowering and senescence. Genotype‐specific extinction coefficients (*k*) were applied to indicate the overall effective LAI. The *k* values were different among cultivars because of the various stem densities, heights, and leaf numbers.

The statistical analysis demonstrated that the maximum LAI varies significantly between cultivars and ecotypes, and the maximum LAI for Alamo is 12, which is much higher than that of CIR (6.57), and the maximum LAI for lowland ecotypes is 11.1, which is much higher than upland ecotype 6 (Figure S2). The maximum LAI value 12 of Alamo is also used in the ALMANCE model (Behrman et al., 2014; Kiniry et al., 1996; Sanderson et al., 1996). The statistical maximum LAI value of CIR 6.57 is similar to the 6.64 found in Iowa, USA (Aurangzaib et al., 2018). Maximum LAI for Alamo of 12 and 7 for CIR is parameterized in the SwitchFor model. The DD factor describes the relationship between LAI incensement along with the DDs during leaf expansion in the SwitchFor model. It was calculated based on measurements from field trials in Iowa (42°0′41″ N, 93°44′34″ W) (Aurangzaib et al., 2018), where daily LAI of the Alamo and CIR was recorded. Together with daily temperature from the National Centers for Environmental Information, linear regression was made between LAI and DD0 and the results showed DD factor is 0.004 for Alamo and 0.006 for CIR (Figure S4). The leaf area decrease along with the DOY during which the leaf senescence was calculated based on the field experiments in two sites Centre Illinois (−88.23, 40.08) (VanLoocke et al., 2012) and “Energy Farm” (40°3′46.209″ N, 88°11′46.0212″ W) (Zeri et al., 2011). The results showed the LAI decrease rate of the Alamo is 0.03 day^−1^ and CIR is 0.049 (Figure S5).

##### Radiation use efficiency

Radiation use efficiency or photosynthesis rate (Pn) are governed by the temperature at which the leaves have formed and the daily temperature, and reduced by drought stress related to the SWD. In the model, the maximum RUE is the theoretical value when crops grow at optimum temperature with no water or nutrient stress. The effective RUE was calculated by using a temperature variation factor (TVF), which was then applied to the maximum RUE. The TVF is a two‐dimensional exponential function that uses the input of the average temperature over the period of leaf formation and the daily temperature at the time of photosynthesis to produce a continuous variable of TVF. Simultaneously, the RUE is affected by soil water deficient with a downregulation algorithm based on the soil water CP (Section 2.1.1).

The statistical analysis of RUE for lowland ecotypes was 3.51 g per MJ IPAR, which was much higher than the upland ecotype 2.32 g per MJ IPAR (Figure S3). The recorded maximum RUE for lowland ecotypes was 5.05 g per MJ for Alamo and the maximum for upland ecotypes was 3.19 g per MJ IPAR of CIR (Kiniry, Johnson, Bruckerhoff, et al., 2011). The maximum RUE used in the ALMANCE (Kiniry et al., 1996; Kiniry et al., 2005) and SWAT model (Trybula et al., 2015) is 4.7, which was measured on the Alamo genotype in TX in 1995–1997 (Kiniry et al., 1999). In this study, the SwitchFor model used a maximum RUE 5.05 for Alamo and 3.19 for CIR, which is consistent with the research of Kiniry et al. (1999) that observed that lowland switchgrass populations had 10 ± 15% greater photosynthetic rates than upland switchgrass populations (Kiniry et al., 1999). Here in this study, the RUE of CIR is 12.5% of the RUE of Alamo.

**FIGURE 4:**
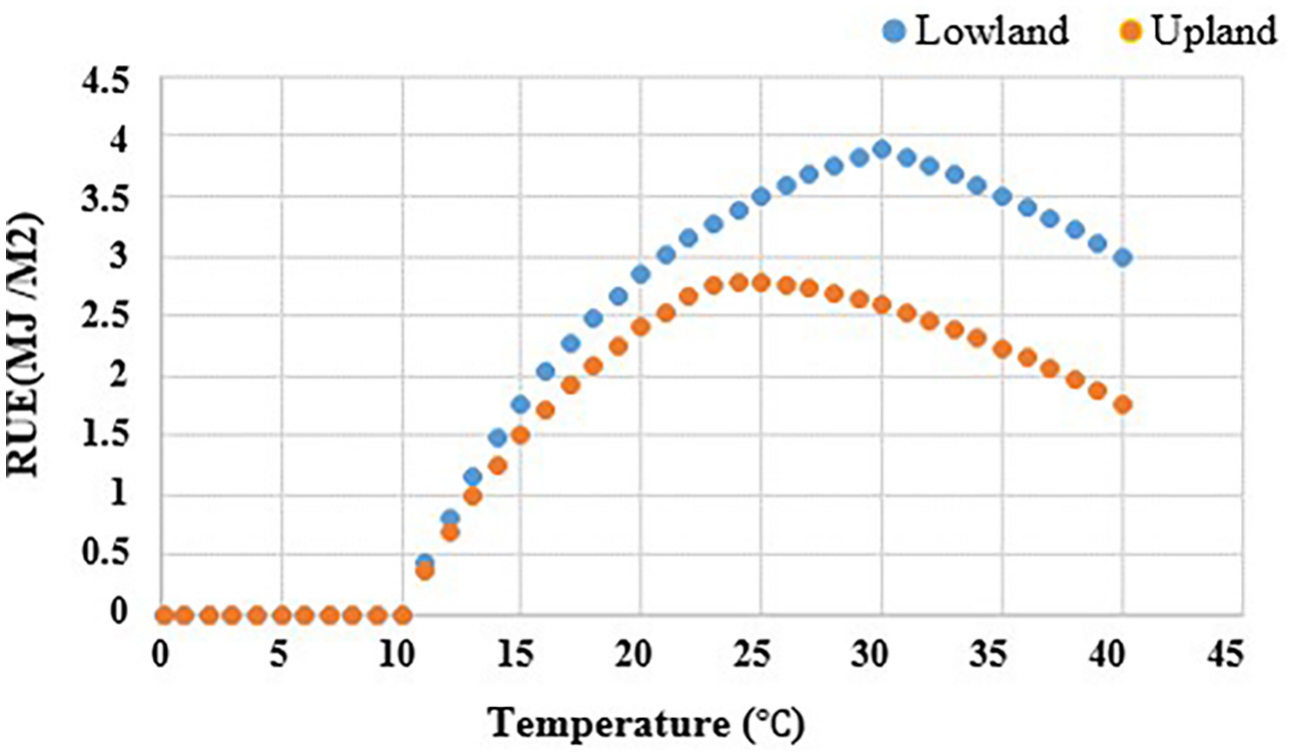
The temperature variation factor of radiation‐use efficiency in SwitchFor model.

Temperature affects growth and development, DM accumulation and partitioning, expansion growth, and phonological development of plants (Kandel et al., 2013). In the SwitchFor model, the RUE is affected by TVF, as described before. The TVF of switchgrass was parameterized based on the published experiment of Liatukas et al. (2015) and Gao et al. (2015). The Pn of the Alamo was measured hourly during the growing season at Ansai (Gao et al., 2015). The Pn increased to the peak at 10:00 and 16:00 in May when the temperature was between 28 and 30°C and was lower between 10:00 and 14:00 when the temperature was between 31 and 33°C (Gao et al., 2015) (Table S2). The regression analysis of leaf Pn against monthly average temperature based on this measurement demonstrated the maximum Pn occurred when the monthly average temperature was 28°C in May. The Pn decreased rapidly when the monthly average temperature was 32°C in June (Figure S6) (Gao et al., 2015). We also summarized all the RUE and its corresponding growing season temperature from the published papers (Table S3). The regression analysis of the RUE against growing season temperature indicated that the maximum RUE occurred when the mean growing temperature was 23°C (Figure S7). Therefore, in this study we used the optimal growing season temperature of 28°C for lowland switchgrass and 23°C for upland switchgrass in the SwitchFor model. The TVF of RUE for lowland and upland switchgrass is shown in Figure 4.

#### 2.1.4 Dry kill and cool kill

In the SwitchFor model, extreme events that result in premature seasonal termination of growth or plant death that requires re‐planting were also estimated. Shoot death (or premature senescence) means that in a given year, the yield is limited but will recover in the following year, while plant death is permanent and crops need to be planted again. The kill event can be caused by either extreme cold or drought. Drought kill occurs when soil moisture below the WP surpasses a time threshold in the growing season. Frost kill occurs when the soil temperature at 10 cm is below a threshold for a specified number of days.

One of the most significant challenges in expanding the productivity zone of different cultivars of switchgrass is the winterkill of varieties not adapted to cold conditions; this highlights the geographical limitation of cultivars (Casler, 2012). It is recommended that cultivars should not be moved outside a range of approximately 500 km of their place of origin (Casler, 2005). The cold tolerance of switchgrass varies between cultivars. The upland ecotypes are usually more cold‐tolerant than the lowland ecotypes. For upland switchgrass, rhizomes survive exposure to temperatures above −20 to −24°C (Sage et al., 2015). The upland ecotype Forestburg, which is rated as having “excellent winter hardness and persistence,” could easily survive in severe winter temperatures of up to −20 to −22°C (Hope & McElroy, 1990). Although the lowland ecotypes usually require a humid and warm environment, Alamo is less tolerant of cold, as observed in the trials on the Loess Plateau. In Yangling, which has a seasonal sub‐humid continental climate, all the upland and lowland ecotypes of switchgrass including 10 cultivars can successfully overwinter and turn green and complete the life cycle. While in the colder site Guyuan, the lowland low‐ecotypes, Alamo and Kanlow, failed to germinate and eventually died. In Dingbian, Alamo and Kanlow grew normally for the establishment year but failed to overwinter. We calculated the number of days in winter when the daily minimum temperature was <0°C in Dingbian, which was 61 days. The US hardness zone map was used to define the plant zone for different switchgrass cultivars based on the origin of the cultivar and the adaptation in the US (Cooney et al., 2017). The hardness zone temperature and the switchgrass cultivars are summarized in Table S4. In the SwitchFor model, the US hardness zone was referenced to parameterize the temperature limitation of switchgrass cultivars. The minimum temperature for lowland switchgrass was −23.3°C and for upland switchgrass −34.4°C. The switchgrass is killed and needed to be replanted when the temperature is below the minimum temperature for 60 days.

#### 2.1.5 Dry matter repartition

There are many different options for harvest timing and frequency, with the two main timings being at peak yield (at anthesis) at the end of the growing season, when the crop can be harvested green, and after completion of senescence, when the crop is dry. For bioenergy and for optimal stand longevity, the dry fully senesced crop is harvested in the spring of the following year (Liatukas et al., 2015). The delayed harvest time allows the plant to translocate nutrients to rhizomes and leaf fall, which results in nutrient maximization (McLaughlin & Kszos, 2005), and allows time for plants to harden for the winter (Douglas et al., 2009). The harvest time also affects the nutrient composition; delaying the harvest from late November to early March can result in reduced ash production and less potassium in the biomass. In the SwitchFor model, at onset of the plant senescence, the peak DM decreases linearly at a certain rate until March the following year when the above‐ground biomass is harvested. The relationship between peak yield and the harvest yield is present as a ratio in the model. The ratio for upland and lowland switchgrass is 0.832, which was determined based on the experiment in Illinois (Heaton et al., 2008).

### 2.2 Sites and data description for model parameterization and validation on the Loess Plateau

Using the parameterization described above, the model was validated by running the SwitchFor model using the soil and meteorological data and comparing the modelled yield to those measured during the field trials. The model performance was evaluated for both upland and lowland ecotypes using the Modval spreadsheet developed by Smith and colleagues (1996).

The field trail data were collected from China, US, Canada, UK, Germany and French which are span in a wider environment and climate condition. There are more than 10 swithcgrass cultivars (Alamo, Blackwell, CIR, Dakota, Forestberg, Illinois USA, Kanlow, Nebraska 28, Pathfinder, and Sunburst) planted in validated sites, and the yield of different ages' switchgrass were measured. The detailed information of the validate sites are shown in Table 3. In these sites, the yearly average yield of each genotype and their seasonal phenology were measured along with the meteorological data. The site‐specific climate data and soil data were used to run the model on each site in each experimental year. Climate input data include the daily‐based minimum temperature, maximum temperature, daily precipitation, evapotranspiration, and solar radiation. For the validation sites in China, the input data were obtained from the meteorological station measurements at the experimental sites, or data from the National Scientific Meteorological Centre (http://data.cma.cn/) when the site meteorological station was not available. The soil data include soil texture (weight of sand, slit, and clay), soil carbon, and bulk density to run the PAWS model (plant available water in soil), and these data were obtained from the field measurements or published paper. The input data of the experimental sites in the United States, Canada, and Europe are obtained from public database. The daily temperature and precipitation data were collected form national canters for environmental information (NOAA), the evapotranspiration data were collected from the National Aeronautics and Space Administration (NASA), and the solar radiation data were collected from Application for Extracting and Exploring Analysis Ready Samples (APPEEARS).

**TABLE 3:** Switchgrass experimental data sets used to validate the model.

Sources	Country	Site name	Latitude (°)	Longitude (°)	Cultivar	Ecotype	Planting year	Harvest year	Measured yield (Mg ha^−1^)	SwitchFor_ modelled yield (Mg ha^−1^)
Arundale et al. (2014)	US	Dekalb	41.85	−88.85	CIR	Upland	2002	2006	7.7	4.9
								2007	6.0	6.0
								2008	10.0	5.9
								2009	5.0	7.1
								2010	4.0	3.7
								2011	10.0	4.1
		Fairfield	38.95	−88.96	CIR	Upland	2004	2006	10.0	13.9
								2007	16.0	16.6
								2008	16.0	14.2
								2009	11.0	16.3
		Orr	39.81	−90.82	CIR	Upland	2004	2006	8.0	7.0
								2007	15.0	9.1
								2008	12.0	13.3
								2009	9.0	13.4
								2010	8.0	10.4
								2011	10.0	10.7
Dohleman et al. (2012)	US	South Farms	40.05	−88.20	CIR	Upland	2002	2006	14.7	14.8
								2007	13.2	12.9
								2008	10.5	11.4
Madakadze, Coulman, et al. (1998)	Canada	McGill University	45.47	−73.75	Multi^a^	Upland	1992	1993	8.8	9.0
								1994	10.2	14.0
								1995	11.8	11.4
								1996	11.1	12.1
Meyer et al. (1994)	US	Red River	46.95	−97.02	Sunburst	Upland	1988	1990	7.3	3.9
								1991	9.3	13.9
								1992	10.3	16.7
Ma et al., (2011) and Yu (2011)	China	Guyuan	36.01	106.27	Multi^b^	Upland	2006	2007	2.6	2.3
								2009	6.1	2.3
								2010	8.0	3.6
		Yangling	34.20	108.11	Multi^b^	Upland	2006	2009	12.6	9.1
								2010	30.2	8.9
		Dingbian	36.82	107.25	Multi^b^	Upland	2009	2009	1.0	0.7
								2010	5.0	0.4
					Alamo, Kanlow	Lowland	2009	2010	1.4	1.3
Provided by Chinese researcher	China	Ansai	36.86	109.32	Alamo	Lowland	2009	2009	1.9	1.9
								2010	2.8	3.2
								2011	4.8	4.8
								2012	7.8	7.0
								2013	7.1	3.1
								2014	6.8	7.2
								2015	4.5	2.2
								2016	4.9	4.3
Christian et al. (2002)	UK	Rothamsted Experimental Station Farm	51.80	−0.35	Kanlow	Lowland	1993	1995	5.5	4.3
								1996	6.2	8.4
								1997	11.6	8.1
								1998	14.0	13.7
Muir et al. (2001)	US	Stephenville	32.22	−98.20	Alamo	Lowland	1992	1992	8.8	15.2
								1993	12.3	13.7
								1994	10.2	13.2
								1995	16.1	18.1
								1996	11.6	11.3
								1997	12.4	12.1
								1998	7.4	6.9
Boehmel et al. (2008)	Germany	University of Hohenheim	48.73	8.93	Kanlow	Lowland	2002	2003	8.0	14.4
								2004	12.0	14.0
								2005	14.0	12.4
Cadoux et al. (2014)	France	INRA experimental station	49.87	3.01	Kanlow	Lowland	2006	2007	19.6	14.0
								2008	18.1	13.5
								2009	17.6	16.9
								2010	14.7	10.9
								2011	14.3	13.4

*Note*: The database from the research of Li et al. (2018) is mainly referred in this table.

^a^ Multi switchgrass cultivars: CIR, New Jersey 50, Blackwell, Shelter, Pathfinder, Sunburst, Forestburg, ND3743, Dakota.

^b^ Multi switchgrass cultivars: Blackwell, Cave‐in‐ Rock, Dakota, Forsberg,Illinois USA, Nebraska 28, Pathfinder, Sunburst.

### 2.3 Spatial analysis of the Loess Plateau region

Using the upland and lowland ecotype parameters, the model was run on a half‐minute of arc grid for the Loess Plateau. The CRU4.04 meteorological data set was extracted for the period 2000–2016 (Harris et al., 2020) and the HWSD soil data (Harmonized Soil Word Database; Global Soil Data Task Group, 2000) used as the input to calculate the harvestable yield in each of the grid cells, which represents a resolution of 1 km. The SwitchFor model was run separately for upland and lowland ecotypes with the ecotype specific parameters. The mean yield, averaged for the period 2006–2016, was used to build opportunity maps of upland and lowland ecotype switchgrass using Arcgis10.5.1. The highest yielding ecotype and its yield were displayed on a 1 × 1 km grid, which was named optimal switchgrass.

The sustainable development of the biomass production was one of the most important considerations, thus the marginal land is the target land that will be used to plant energy crops in this study. The marginal land, which has been defined and identified on the Loess Plateau in the research of Liu et al. (2021), was adopted in this study. In the research of Liu et al. (2021), the marginal lands were defined in two land‐use scenarios in terms of the different grassland use. Extra consideration of the slope was added to the marginal identification in this study so that the land with the slope greater than 15° was excluded. The terms marginal land use scenario 1 and marginal land use scenario 2 was marginal land that excludes a slope greater than 15°.

## 3 RESULTS

### 3.1 Model validation

**FIGURE 5:**
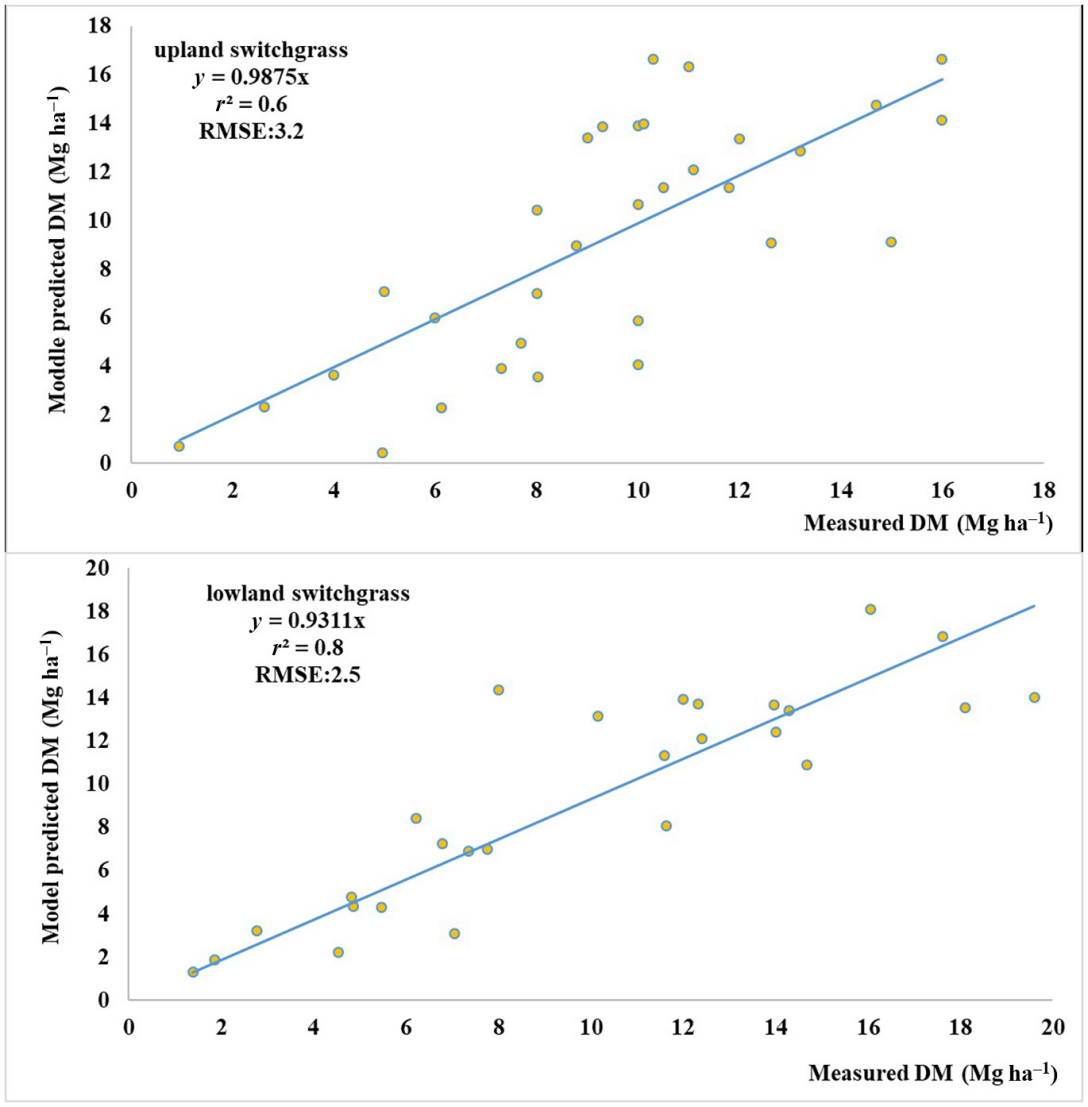
The regression of the modelled dry matter and actual measurement on the Loess Plateau. (a) Upland switchgrass; (b) lowland switchgrass.

Tables 3 shows the modelled DM yield from SwitchFor model and the actual measurements of switchgrass for each ecotype in each year across all the field trail sites. To compare the simulated DM yield with the actual measurements, a linear regression of the simulated DM yield with the actual measurement of upland and lowland switchgrass of all samples is displayed in Figure 5. The *r* ^2^ (coefficient of determination) is 0.6 and 0.8 and root‐mean‐squared error (RMSE) is 3.3 and 2.5 for upland and lowland switchgrass separately which demonstrates that the SwitchFor model could predict switchgrass yield well. Compared with upland switchgrass, the higher *r* ^2^ and lower RMSE of lowland switchgrass indicates that the model could predicate the yield of lowland switchgrass better than upland switchgrass.

In addition, the sites of validation samples span latitude from 32.22° to 51.80° and longitude from −98.20° to 109.32° that the environment and climate condition is diverse. The measured upland and lowland ecotypes in the validated sites include a variety of the switchgrass cultivars. Moreover, the yield of different ages' switchgrass was measured. The good validation results proved that the SwitchFor model could capture the GEI, and thus give a good prediction of the switchgrass yield in a wide environment and climate condition.

### 3.2 Spatial yield map of upland and lowland switchgrass

**FIGURE 6:**
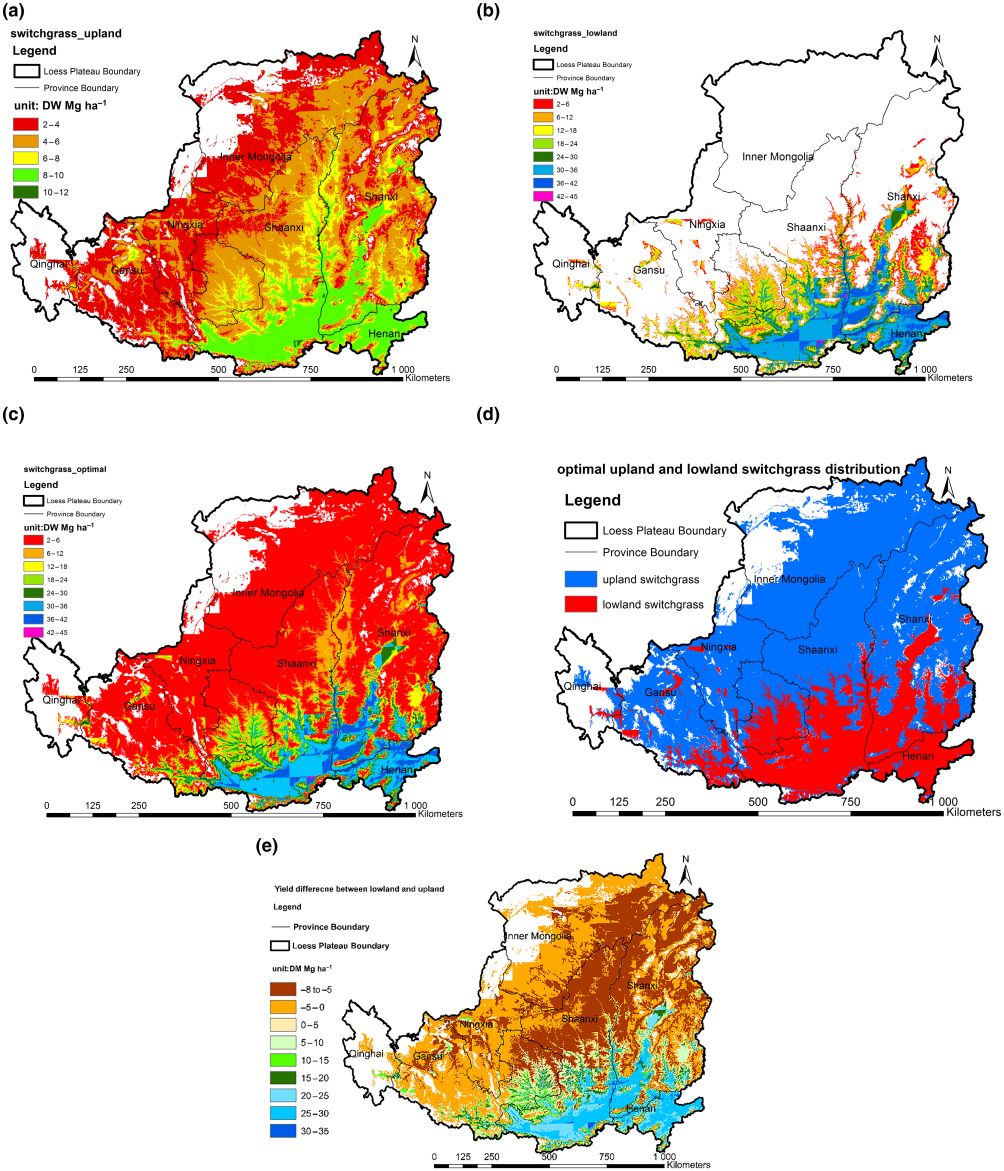
The spatial distribution map of switchgrass biomass yield across the Loess Plateau. (a) Upland switchgrass; (b) lowland switchgrass; (c) optimal switchgrass; (d) optimal switchgrass ecotype; (e) yield difference between lowland and upland switchgrass (a negative number indicates that the yield of lowland switchgrass is lower than upland switchgrass).

Figure 6a,b shows the yield spatial distribution of upland and the lowland switchgrass on the Loess Plateau separately. The upland and lowland switchgrass have different distribution preferences. Compared with the lowland switchgrass, the upland switchgrass has a wider distribution that it could survive in 78% of the Loess Plateau, except a small area including the northwest area in Inner Mongolia, a small area in Ningxia, and the most area of the Qinghai. The lowland switchgrass is mostly distributed on south and southeast of the Loess Plateau such as the southern Shaanxi, southern Shanxi, and Henan province where the climate is warmer and moister. The distribution area of lowland switchgrass is 34% of that of upland switchgrass, accounting for 27% of the total area of the Loess Plateau. These results are consistent with the previous research that found that upland ecotypes are able to adapt to conditions across a wide geographical region (Casler et al., 2004; Casler & Boe, 2003; Hopkins et al., 1995). It should be noted that the description of provinces in this text only refer to the part that is in the range of the Loess Plateau, since the Loess Plateau includes only part of the provinces (Figure 1).

The yield distribution trend is found to be similar for both upland and lowland switchgrass that the yield increased from northwest to southeast (Figure 6a,b); however, yield in each grid cell varies significantly between upland and lowland. The yield difference between lowland switchgrass and upland switchgrass is in the range of 0–34 Mg ha^−1^ (Figure 6d). The lowland switchgrass obtains much higher yield over upland switchgrass in south and southeast parts of the Loess Plateau where both upland and lowland switchgrass can adapt. For the north and northwest regions where it is colder or dryer, the lowland switchgrass could not withstand the conditions, and only the upland switchgrass could survive (Figure 6e). The average yield of the lowland switchgrass is much higher than the upland switchgrass, with lowland switchgrass at around 22 Mg ha^−1^ and upland switchgrass 5 Mg ha^−1^ (Table 4). Despite the lower average yield of the upland switchgrass, the total yield without considering the land availability did not have much difference between upland and lowland switchgrass owing to the wider distribution of the upland switchgrass (Table 4). We compared the yield of the upland and lowland switchgrass in each 1 × 1 km grid cell and extracted the higher yield ecotype as the optimal ecotype, and generated a switchgrass optimal yield map. The total optimal yield without considering the land availability is 547.6 Tg, which is 1.4 times and 1.3 times of the individual lowland or upland switchgrass, respectively (Figure 6c; Table 4). It demonstrates that estimating the yield without considering the ecotype or cultivars might underestimate the yield potential, since a single cultivar has a limited adaptation zone compared with the species as a whole due to the strongly photoperiodic nature of switchgrass and apparent genetic variation for heat and cold tolerance (Casler, 2005). A combined analysis of different switchgrass cultivars can extend the possible adaptation zone of the switchgrass in a region by making full use of the land with the plantation of the most suitable cultivar.

**TABLE 4:** The yield of the switchgrass estimated by the SwitchFor model.

	Total biomass (Tg)	Area (M ha)	Area percentage (%)	Average yield (Mg ha^−1^)	Standard error (Mg ha^−1^)
	Lowland switchgrass				
All land	381.6	17.18	26.8^a^	21.6	12.1
Marginal land use scenario 1^b^	59.7	2.90	14.0^c^	19.9	11.5
Marginal land use scenario 2^b^	30.8	1.67	12.5^c^	18.7	11.8

	Upland switchgrass				
All land	419.4	50.18	78.3^a^	5.2	2.0
Marginal land use scenario 1^b^	63.6	11.93	57.4^c^	4.6	1.8
Marginal land use scenario 2^b^	39.7	7.54	58.9^c^	4.5	1.8

	Optimal switchgrass				
All land	547.6	50.25	78.4^a^	10.0	10.6
Marginal land use scenario 1^b^	106.4	12.60	60.6^c^	7.3	8.4
Marginal land use scenario 2^b^	61.6	7.88	61.6^c^	6.7	7.6

*Note*: The area of the Loess Plateau is 64.08 Mha. The area of the marginal land use scenario 1 is 20.8 Mha, and the area of the marginal land use scenario 2 is 12.8 Mha.

^a^ The percentage is the ratio of switchgrass distribution area estimated by the SwitchFor model to the total Loess Plateau area.

^b^ The definition and the identification of the marginal land can be found in research of Liu et al. (2021).

^c^ The percentage is the ratio of switchgrass area estimated by the SwitchFor model to the total corresponding marginal land area.

**FIGURE 7:**
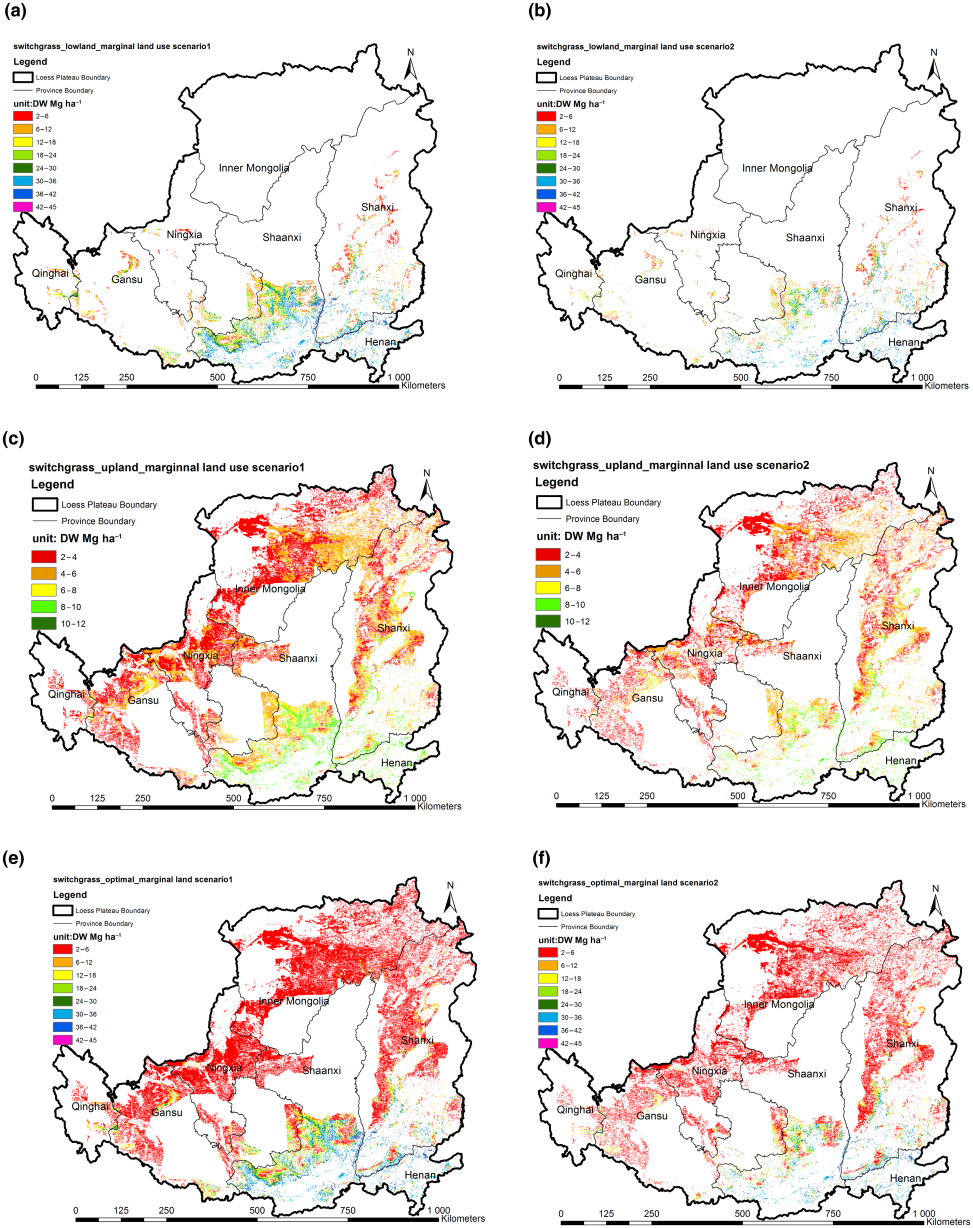
The spatial distribution of switchgrass biomass yield on the available marginal land of the Loess Plateau. (a) Upland switchgrass in marginal land use scenario 1; (b) upland switchgrass marginal in land use scenario 2; (c) lowland switchgrass marginal in land use scenario 1; (d) lowland switchgrass marginal in land use scenario2; (e) optimal switchgrass marginal in land use scenario 1; (f) optimal switchgrass marginal in land use scenario 2.

Figure 7 shows the switchgrass spatial distribution on the marginal land of the Loess Plateau. The yield potential on the marginal land of the lowland switchgrass is 30.8 to 59.7 Tg (Figure 7a,b) and upland switchgrass is 39.7–63.6 Tg (Figure 7c,d). There is not much difference in total yield potential between upland and lowland switchgrass, although the average yield of lowland switchgrass (20 Mg ha^−1^) is much higher than that of upland switchgrass (5 Mg ha^−1^). The optimal yield on the marginal land of the Loess Plateau is 61.6–106.4 Tg (Figure 7e,f), which is about twice yield potential of the individual ecotype (Table 4).

## 4 DISCUSSION

### 4.1 Uncertainty and limitation of this study

There are some uncertainties and limitation in this study. Upland and lowland ecotypes have generally been differentiated on the basis of plant phenotype that lowland plants are taller, have fewer and larger tillers, longer and wider leaves, thicker stems, and are later in flowering than upland plants which result in the higher yield of the lowland switchgrass (Casler, 2012). The upland and lowland ecotypes, respectively, contain a large variety of cultivars, and the cultivars of the same ecotype also have different phenotype and adopt zone amongst which may results to different yield potential. In this study, only upland and lowland switchgrass were parameterized because of the limited cultivar‐specific plant growth data. For some of the key parameters, such as *k*, RUE, and the maximum LAI, the values are the results of statistical analyses of values of many cultivars of the same ecotype; the summarized values of a parameter are also different among the cultivars with the same ecotype. Once the upland and lowland switchgrass was considered, the total potential yield was estimated at twice that of the individual cultivar. If more cultivar‐specific data were available, it would give more precise yield estimation.

Parameterising the DDs for physiostats has some uncertainty. The DDs of physiostats is a particularly important parameter in the SwitchFor model; however, the DDs vary significantly in different locations. The Alamo originated from 28° N, but when moved to 37° N, it required 350 DD and 685DD for leaf emergence and internode development, respectively. When moved to 32° N, it required 430 DD and 1020 DD. The CIR originated from 38° N, but when moved to 37° N, it required 200 DD for leaf emergence and 378 DD for internode development. When moved to 32° N, it required 350 DD and 550 DD for leaf emergence and internode development, respectively. The duration of the vegetation period of both Alamo and CIR was 300 DDs shorter at Blacksburg (37° N) then Stephenville (32° N). They both had a longer vegetation period and matured earlier when moving southward because of the earlier emergence in spring and exposure to shorter day length in low latitude, whereas moving northward they had short vegetation periods but long reproductive periods (Sanderson & Wolf, 1995). Giannoulis et al. (2016) demonstrated that the “optimum” latitude for upland ecotypes is between approximately 36°and 39° where the maximum yield emerged, and below and above which both maximum and minimum yields were lower. There was no clear relationship between latitude and the DDs and thus yield. Therefore, in this study, we used the average DD from all the DDs summarized from the published papers and the location on the Loess Plateau. A sensitivity analysis was also conducted to determine 10°C (Figure 3) as the best base temperature to calculate the DD in the SwitchFor model. With more research on the relationship of DDs and physiostats, the model will become more improved.

### 4.2 Comparison of the SwitchFor with fuzzy logical models

The fuzzy logical model was previously developed and validated on the marginal land of the Loess Plateau and used to predicted the yield of the switchgrass (Liu et al., 2021). We made a comparison of the yield of the switchgrass estimated from the SwitchFor model developed in this study with results from the fuzzy logical model.

**FIGURE 8:**
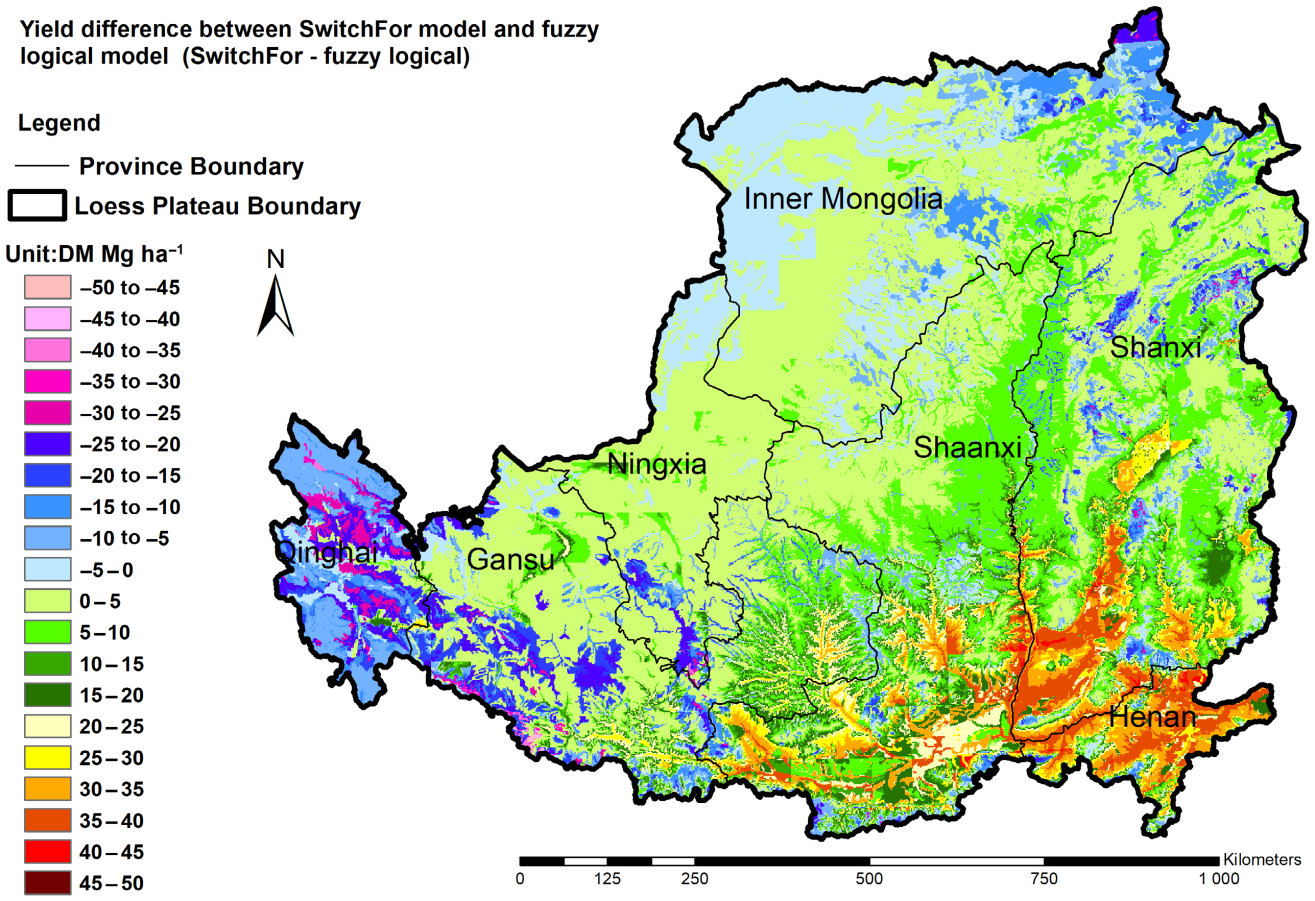
Switchgrass yield difference between the SwitchFor model and the fuzzy logical model.

The fuzzy logical model was previously developed to estimate the land suitability and yield of the switchgrass on the marginal land of the Loess Plateau; it is a general switchgrass model in which the ecotype or cultivars are not considered (Liu et al., 2021). The total yield of the switchgrass in marginal land of the Loess Plateau was estimated to be 39.9–65.8 Tg according to the fuzzy logical model (Figure S8; Table S5), which is not very different to the total yield of individual upland and lowland from the Switchfor model (Table 4). However, the total optimal yield from the SwitchFor model is about twice of the total yield from fuzzy logical model. Figure 8 shows the different yield of the optimal yield generated from the SwitchFor model and the general yield from fuzzy logical model. For the most of par, the difference is between −10 and 10 Mg ha^−1^. The most significant difference occurs in the the southeast of the region of the Loess Plateau where the lowland switchgrass is more yielding than upland switchgrass from the results projected by SwitchFor. However, the yield estimated by the fuzzy logical model in the southeast of the region is more like the results of upland switchgrass projected by the SwitchFor model. Due to fact that the climate in the southeast part of the Loess Plateau being warmer and moister where the lowland switchgrass grows well and can obtain higher yield than upland switchgrass. Yangling, for example, is in the southeastern part of the Loess Plateau, and the field trials demonstrated that the yield of lowland switchgrass Alamo was almost twice of the upland switchgrass CIR (Ma et al., 2011). This indicates that the general fuzzy logical model might underestimate the total yield potential. The optimal yield from the cultivar‐specific SwitchFor model can reflect the actual situation well through comprehensively analysing the cultivation‐specific adoption preference and the yield.

### 4.3 Compare the biomass potential of the switchgrass and other potential plant species

There are many different energy crops hold potential to develop biomass‐derived industry. These include perennial C4 grasses such as Miscanthus and switchgrass, short rotation coppices such as poplar and willow, and an oil‐producing shrub, *Jatropha* (Sang & Zhu, 2011). In this study, switchgrass is mainly investigated on the Loess Plateau region, while this does not mean that the switchgrass should be planted on overall marginal land. Because planting a single variety in a large area can easily lead to negative impact on biodiversity, multiple energy crops should be planted on the most suitable sites.

**FIGURE 9:**
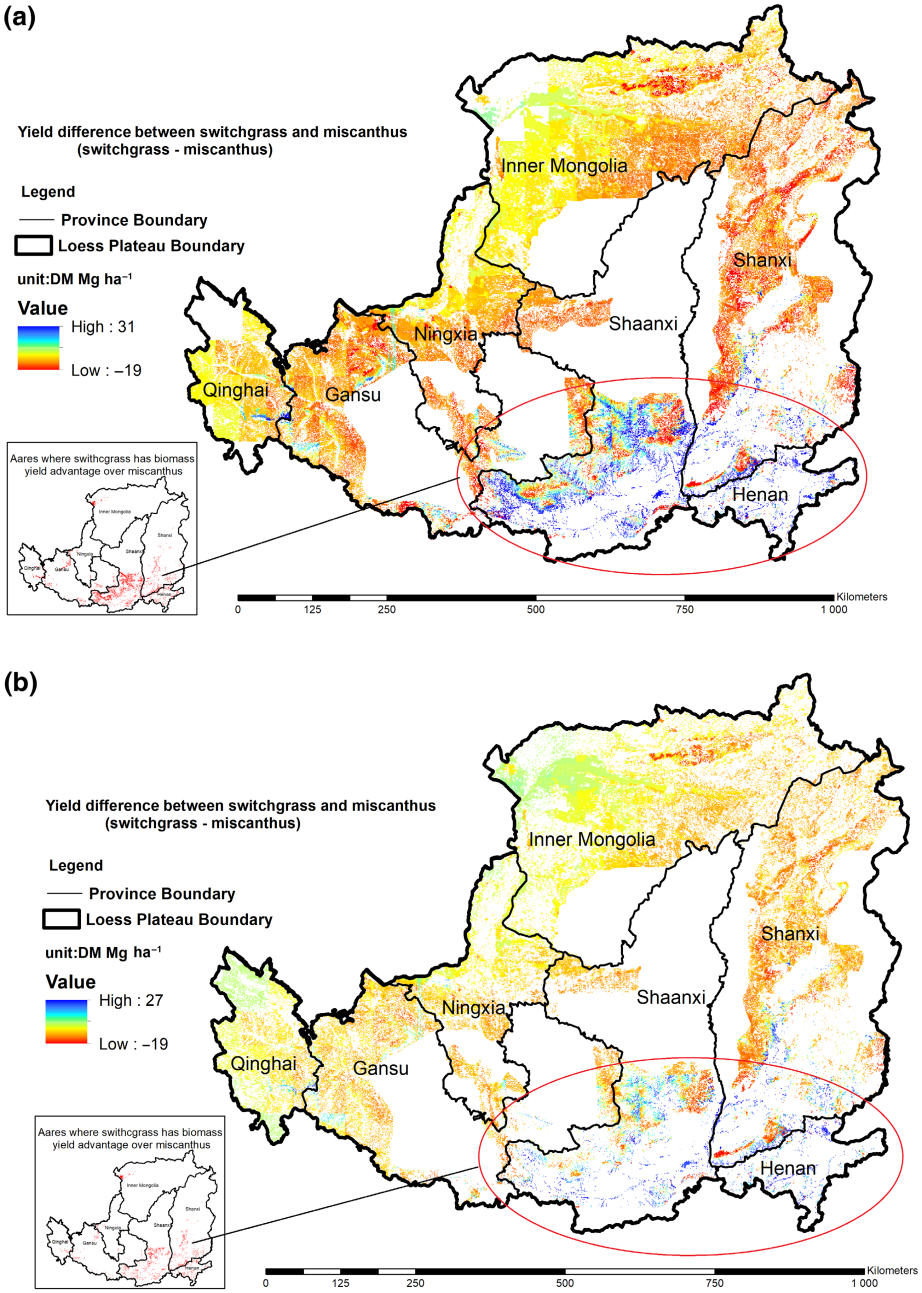
The biomass yield difference between switchgrass and miscanthus on the marginal land of the Loess Plateau. (a) Marginal land use scenario 1; (b) marginal land use scenario 2.

Zhang et al. (2020) estimated miscanthus yield potential in China using MiscanFor model. We extracted the miscanthus spatial distribution yield on Loess Plateau region by overlaying the marginal land maps (scenario1 and scenario2) of Loess Plateau used in this study. Figure 9 shows the yield difference of switchgrass and miscanthus by using optimal switchgrass yield generated from Switchfor model minus the miscanthus yield from MiscanFor model in each grid cell. The total yield of miscanthus is about 139–222 Tg, which is much higher than total yield of switchgrass. While the switchgrass has yield advantage over miscanthus in the southern Loess Plateau. If the switchgrass is planted in where it has higher yield than miscanthus, the total yield of the biomass will increase 11–21 Tg. Willow and poplar are also considered promise as a feedstock for biofuels on the marginal land. Especially, poplar is used for landscape and agriculture use as well as windbreaks and shelterbelts on the Loess Plateau (Pleguezuelo et al., 2014). The investigation of the potential of poplar and willow as energy crops to produce bio‐energy has never been conducted on the Loess Plateau and need to be done in the future. Liang et al. (2006) investigated on water consumption characteristics and water use efficiency of poplar under SWDs on the Loess Plateau. The study showed that the poplar may not suitably planted in the region of Loess Plateau on a large scale because of the large water consumption, and it could be cultivated in shade valleys and near cave roads. Consequently, to develop biomass‐derived industry, the comprehensive analysis of environment and socio‐economic of multi‐energy crops should be conducted in the future.

## 5 CONCLUSION

Owing to the different adaption and phenotypes between upland and lowland switchgrass, a cultivar‐specific SwitchFor model was developed by incorporating the key parameters from a well‐developed MiscanFor model. The model was validated in a wide environment and the good validation results demonstrated that the Switchfor model could capture the GEI and thus give an accurate prediction of the swithcgrass in a wider region. The SwitchFor model was applied to conduct a spatially explicit evaluation of the biomass yield of switchgrass on the marginal land of the Loess Plateau. The results demonstrated that the upland and lowland ecotypes differed significantly in spatial distribution of the yield and adaptation zone. The optimal switchgrass map was generated by comprehensive analysis of the upland and lowland switchgrass yield distribution which significant improvement in terms of producing a more accurate yield prediction.

Overall, this study provides a well‐developed cultivar‐specific growth model for switchgrass. Moreover, the methodology framework could be further adopted to parameter more switchgrass cultivars with more field trial data of different cultivars available. A comprehensive analysis of multi cultivars will provide more detailed information for farmers and land managers on cultivar selection in specific sites, as well as a more accurate prediction of the biomass yield potential for the government and investors to make long‐term land use and sustainable bio‐energy development strategy.

## Supporting information


Data S1

## Data Availability

The climate data of China was obtained from the National Scientific Meteorological Centre (http://data.cma.cn/). The climate data of the US, Canada, and European was obtained from public database. The daily temperature and precipitation data was obtained from national centers for environmental information (NOAA), the evapotranspiration data were collected from the National Aeronautics and Space Administration (NASA), and the solar radiation data were collected from Application for Extracting and Exploring Analysis Ready Samples (APPEEARS). The key output of SwitchFor model in this study are the spatial distribution biomass yield maps of switchgrass (upland switchgrass, lowland switchgrass, and optimal switchgrass) on the marginal land of the Loess Plateau in the land use scenario1 and scenario2. The data supporting the findings of this study are openly available in the file name of “Spatial distribution of the yield biomass of switchgrass on the marginal land of the Loess Plateau” at https://doi.org/10.6084/m9.figshare.21047224.v1. The data are raster maps in Tiff formation with resolution of 1 × 1 km^2^.
