# Supplementary material for: The development of a new crop growth model SwitchFor for yield mapping of switchgrass
Source: Glob Change Biol Bioenergy. 2022 Oct 3;14(12):1281–302. doi: 10.1111/gcbb.12998 (PMC9828430; doi:10.1111/gcbb.12998)
Supplement: Supplementary file 1 — Data S1 [file GCBB-14-1281-s001.docx]

**Supplement**

1. **Physiosates in SwitchFor model**

Onset of the shoots start after the end of the dormancy. This stage is triggered when thermal time (expressed as degree days) is above a threshold, the temperature is above the shoot emergence temperature, photoperiod is above a threshold, and SWC is above CPstop. The last frost in the spring will kill the shoots and the process will restart. After the shoot emergence, leaf expansion and the photosynthesis start. This stage is triggered when DD is above a threshold, the temperature is above the photosynthesis temperature and shoot emergence temperature, and the SWC is above the CPstop. At this stage, the leaf expansion and the photosynthesis start at a rate defined by SWC and temperature. Leaves continue expanding to the maximum LAI or until one of the following, conditions occur that stops the leaf expansion and trigger the leaf senescence. These conditions are DD above a threshold, first frost, the temperature above a temperature threshold that result in overheating, cold (x days below temperature threshold) or drought conditions (x days below CPstop). At leaf senescence stage, leaf area stops expansion but photosynthesis continues at a rate dependent upon temperature and SWC. The DM continue to accumulate until plant start of senescence. Peak harvest is calculated at this time. The Plant senescence is triggered when DD is above a threshold, earlier cold (continuous days’ temperature below a temperature threshold), first frost day, or dry (continuous days’ soil water below CPstop). In plant senescence stage, leaf area decreases and plant flowers and sets seed, photosynthesis declines. The plant goes into the plant drying stages. At this point photosynthesis stop, leaves drop and nutrients repartition to root and rhizome. All rates triggers and brakes are species and genotype dependent. Genotypes are day length sensitive then this parameter is added to the start and stop of the growth period.

**Table S1. Literature review of degree-days of Physiostat and calculated degree-days based on the field trail**

| Source | Location |  |  | Stage0 | Stage1(shoot) | Stage2(leaf ) | Stage3(LAI max) | Stage4(DM max) | Stage5 |
| --- | --- | --- | --- | --- | --- | --- | --- | --- | --- |
| Gao et al. (2015) and GAO et al. (2017) | Ansai, China (36°51′30″N,109°19′23″E) | Lowland | DOY | - | 100 | 112 | 213 | 248 | 263 |
|  |  |  | DD10 | - | 83 | 137 | 1068 | 1422 | 1520 |
|  |  |  | DD1 | - | 347 | 477 | 2275 | 2936 | 3188 |
|  |  |  | DD0 | - | 393 | 534 | 2432 | 3128 | 3398 |
|  | Yangling, China  (34°12′N,108°7′E) |  | DOY | - | 95 | 107 | 209 | 244 | 263 |
|  |  |  | DD10 | - | 107 | 163 | 1348 | 1807 | 1972 |
|  |  |  | DD1 | - | 432 | 568 | 2657 | 3431 | 3766 |
|  |  |  | DD0 | - | 489 | 637 | 2828 | 3637 | 3991 |
|  | Dingbian, China  (36°49′N,107°15′E) |  | DOY | - | 102 | 114 | 218 | 253 | 265 |
|  |  |  | DD10 | - | 79 | 128 | 1100 | 1399 | 1475 |
|  |  |  | DD1 | - | 349 | 473 | 2330 | 2932 | 3109 |
|  |  |  | DD0 | - | 389 | 524 | 2484 | 3122 | 3310 |
|  | Guyuan, China  (36°0′N,106°16′E) |  | DOY | - | 105 | 117 | 222 | 255 | 263 |
|  |  |  | DD10 | - | 55 | 83 | 807 | 978 | 1016 |
|  |  |  | DD1 | - | 286 | 379 | 1977 | 2436 | 2533 |
|  |  |  | DD0 | - | 332 | 434 | 2136 | 2627 | 2732 |
| Aurangzaib et al. (2018) | Lowa ,US  (42◦0′41″ N, 93◦44′34″ W) | Lowland | DOY | - | 110 | 123 | 198 | 263 | - |
|  |  |  | DD10 | - | 156 | 214 | 935 | 1530 | - |
|  |  |  | DD1 | - | 436 | 583 | 1911 | 3086 | - |
|  |  |  | DD0 | - | 484 | 643 | 2045 | 3286 | - |
|  |  | Upland | DOY | - | - | - | 191 | 232 | - |
|  |  |  | DD10 | - | - | - | 838 | 1282 | - |
|  |  |  | DD1 | - | - | - | 1771 | 2610 | - |
|  |  |  | DD0 | - | - | - | 1899 | 2779 | - |
| Heaton, Dohleman, and Long (2008) | Centre Illinois, US  (40.08 N, 88.23 W) | Upland | DOY | - | - | - | 220 | 228 | - |
|  |  |  | DD10 | - | - | - | 1173 | 1260 | - |
|  |  |  | DD1 | - | - | - | 2372 | 2531 | - |
|  |  |  | DD0 | - | - | - | 2526 | 2694 | - |
| Sanderson and Wolf (1995) | Stephenville, US  (32°13' N, 98°12' W) | Lowland | DOY | - | 60 | 75 | 197 | 227 | 245 |
|  |  |  | DD10 | - | 134 | 218 | 1540 | 2006 | 2247 |
|  |  |  | DD1 | - | 441 | 631 | 3000 | 3736 | 4139 |
|  |  |  | DD0 | - | 488 | 692 | 3180 | 3946 | 4367 |
|  |  | Upland | DOY | - | - | - | 139 | 200 | 214 |
|  |  |  | DD10 | - | - | - | 745 | 1591 | 1808 |
|  |  |  | DD1 | - | - | - | 1685 | 3078 | 3420 |
|  |  |  | DD0 | - | - | - | 1807 | 3261 | 3618 |
|  | Blacksburg, US  (37°11' N, 80°25' W) | Lowland | DOY | - | 100 | 123 | 215 | 248 | 273 |
|  |  |  | DD10 | - | 60 | 156 | 827 | 1092 | 1261 |
|  |  |  | DD1 | - | 352 | 602 | 2035 | 2595 | 2976 |
|  |  |  | DD0 | - | 407 | 678 | 2203 | 2796 | 3202 |
|  |  | Upland | DOY | - | - | - | 188 | 220 | 244 |
|  |  |  | DD10 | - | - | - | 532 | 864 | 1060 |
|  |  |  | DD1 | - | - | - | 1497 | 2117 | 2526 |
|  |  |  | DD0 | - | - | - | 1638 | 2290 | 2723 |
| Van Esbroeck, Hussey, and Sanderson (1997) | TX,US  (30°38′N,96°20′W) | Lowland | DOY | - | 60 | 72 | 195 | 225 | 243 |
|  |  |  | DD10 | - | 209 | 282 | 1832 | 2353 | 2413 |
|  |  |  | DD1 | - | 591 | 750 | 3360 | 4150 | 4150 |
|  |  |  | DD0 | - | 642 | 813 | 3544 | 4364 | 4364 |
|  |  | Upland | DOY | - | - | - | 137 | 198 | 212 |
|  |  |  | DD10 | - | - | - | 884 | 1369 | 2137 |
|  |  |  | DD1 | - | - | - | 1890 | 3443 | 3818 |
|  |  |  | DD0 | - | - | - | 2016 | 3630 | 4019 |

1. **Literature review and statistical analysis of key parameters**

**2.1 Statistical analysis of k, LAI and RUE**


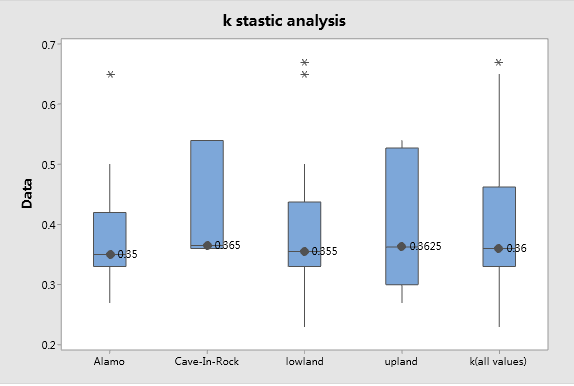


**Fig S1. The statistical analysis of k value**


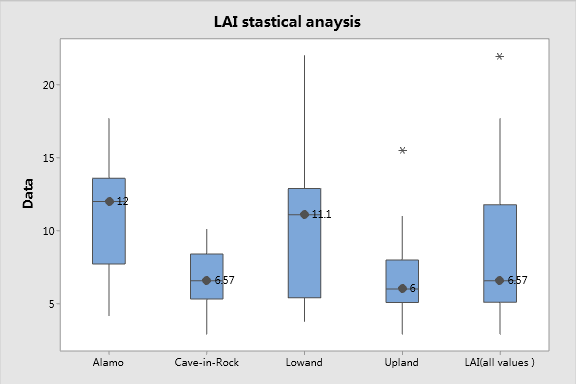


**Fig S2. Statistical analysis of LAI**

**
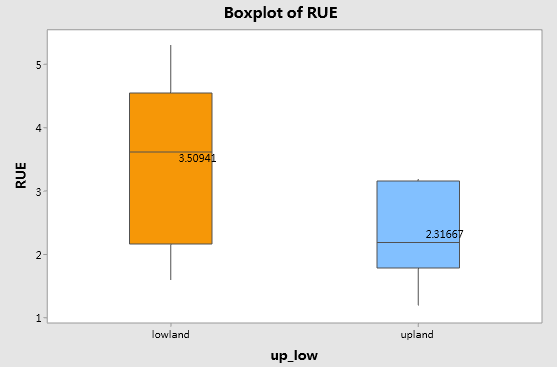
**

**Fig S3. The statistical analysis of k value**

**2.2 DD factor**

**
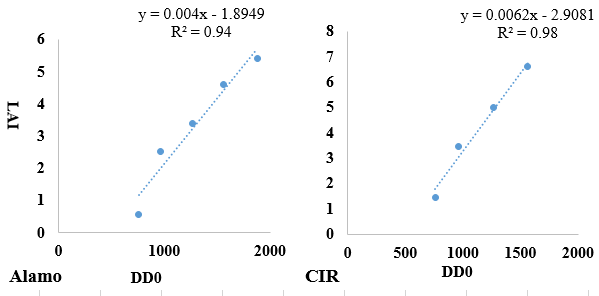
**

**Fig S4. DD factor of Alamo and Cave In Rock**

**2.3 LAI dec**

**
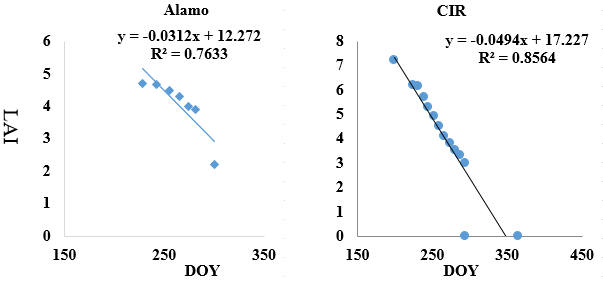
**

**Fig S5. LAI dec of Alamo and Cave In Rock**

**2.3 RUE vs growing season temperature**

**Table S2. The photosynthesis rate (Pn) variation of Alamo against daily temperature in May, Jun, August and September**

| Time of the day | May | | Jun | | August | | September | |
| --- | --- | --- | --- | --- | --- | --- | --- | --- |
|  | T （℃） | Pn | T（℃） | Pn | T（℃） | Pn | T（℃） | Pn |
| 8 | 23.61 | 16.34 | 28.00 | 7.67 | 19.05 | 9.13 | 17.59 | 7.05 |
| 10 | 27.68 | 22.31 | 33.40 | 14.26 | 26.79 | 15.35 | 24.57 | 11.94 |
| 12 | 31.35 | 18.40 | 34.37 | 12.06 | 30.33 | 16.93 | 24.21 | 11.69 |
| 14 | 33.05 | 19.86 | 34.59 | 13.27 | 28.12 | 11.94 | 23.47 | 9.61 |
| 16 | 31.06 | 21.46 | 33.20 | 11.07 | 27.46 | 14.00 | 24.94 | 11.58 |
| 18 | 25.45 | 9.75 | 29.58 | 7.55 | 26.93 | 3.52 | 17.06 | 3.75 |
| Average | 28.70 | 18.02 | 32.19 | 10.98 | 26.45 | 11.81 | 21.97 | 9.27 |

**Table S3. Growing season temperature and RUE**

| Source | Establishment year | sites and year | Growing season temperature(April – September ) | RUE | |
| --- | --- | --- | --- | --- | --- |
|  |  |  |  | Alamo | CIR |
| (James Robert Kiniry et al. 2011) | 2007 | Mo-2008 | 20.34 | 5.05 | 3.15 |
|  | 2007 | Mo-2009 | 19.88 | 3.56 | 3.19 |
|  | 2007 | TX-2008(irrigated ) | 25.29 | 1.96 |  |
|  | 2007 | TX-2009(irrigated ) | 25.07 | 3.04 |  |
|  | 2007 | TX-2010(irrigated ) | 26.39 | 3.35 |  |
|  | 2007 | TX-2008(no-irrigation ) | 25.29 | 1.97 |  |
|  | 2007 | TX-2009(no-irrigation ) | 25.07 | 2.07 |  |
|  | 2007 | TX-2010(no-irrigation) | 26.39 | 2.26 |  |
| (J. R. Kiniry, Tischler, and Van Esbroeck 1999) | 1992 seedling | TX-1995 | 25.26 | 4.00 |  |
|  | 1992 seedling | TX-1996(drought stress) | 26.16 | 4.00 |  |
|  | 1992 seedling | TX-1997 | 23.30 | 5.30 |  |
|  | 1993 seeds | TX-1995 | 25.26 | 4.40 |  |
|  | 1993 seeds | TX-1996(drought stress) | 26.16 | 1.60 |  |
|  | 1993 seeds | TX-1997 | 23.30 | 5.00 |  |
| (Madakadze et al. 1998) | 1995 | Canada-1996 | 15.26 |  | 2.38 |

**Figure S6. The growing season temperature VS leaf photosynthetic rate (Pn) of Alamo in Ansai**

**FigureS7. The growing season temperature vs RUE from literature review**

**2.4 Cold kill**

**Table S4. Overwinter temperature ranges of Switchgrass cultivars based on the suggested US hardness zone**

|  | Hardness zones | The average minimum temperature (°C) |
| --- | --- | --- |
| Alamo | 6.7.8.9 | -23.3 **~** -1.1 |
| Kanlow | 5.6.7.8 | -28.9 **~** -6.7 |
| CIR | 4.5.6 | -34.4 **~** - 17.8 |
| Blackwell | 5.6.7.8 | -28.9**~** -6.7 |
| Trailblazer | 4.5 | -34.4**~** 23.3 |
| Sunburst | 3.4.5 | -40 **~** -23.3 |
| Summer | 4.5 | -34.4**~** -23.3 |
| Shawnee | 5.6.7 | -28.9**~** -12.2 |

**3. Fuzzy logical model**

**Table S5. The yield of the switchgrass estimated by the Fuzzy logical model**

| Land type | Total biomass (Tg) | Average yield(Mg ha^-1^) | STD(Mg ha^-1^) |
| --- | --- | --- | --- |
| All land | 244.2 | 13.4 | 7.3 |
| Marginal land scenario1 | 65.8 | 13.3 | 7.5 |
| Marginal land scenario2 | 39.9 | 12.9 | 7.7 |


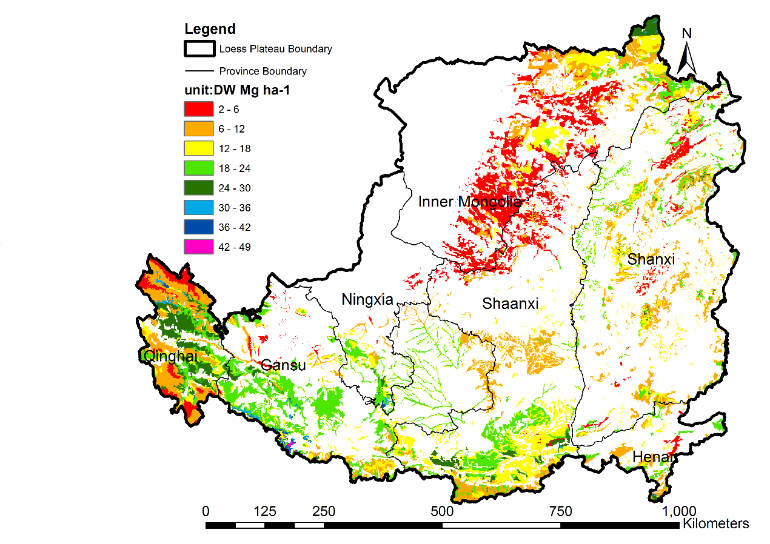
**(a)**

**(b)**

**
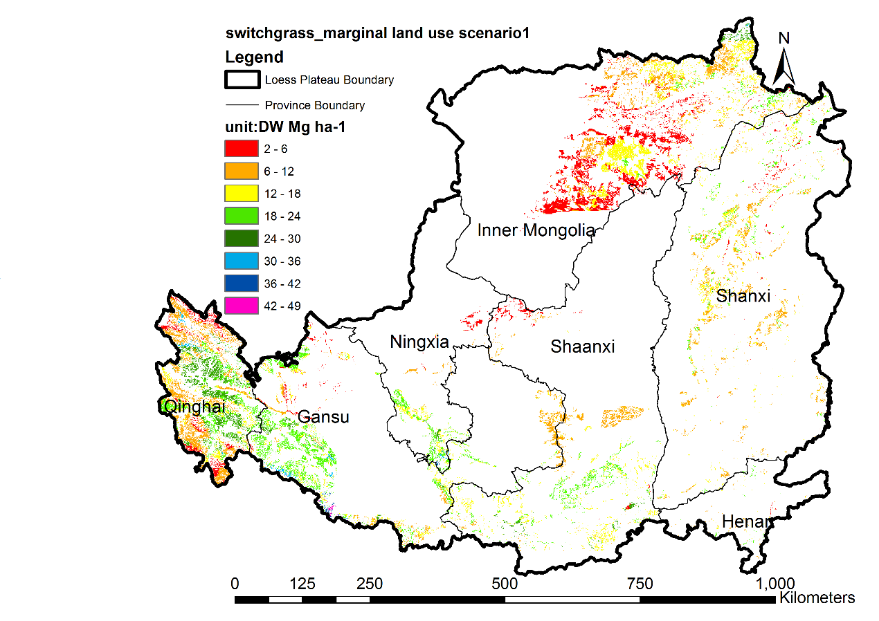
**

**(c)**

**
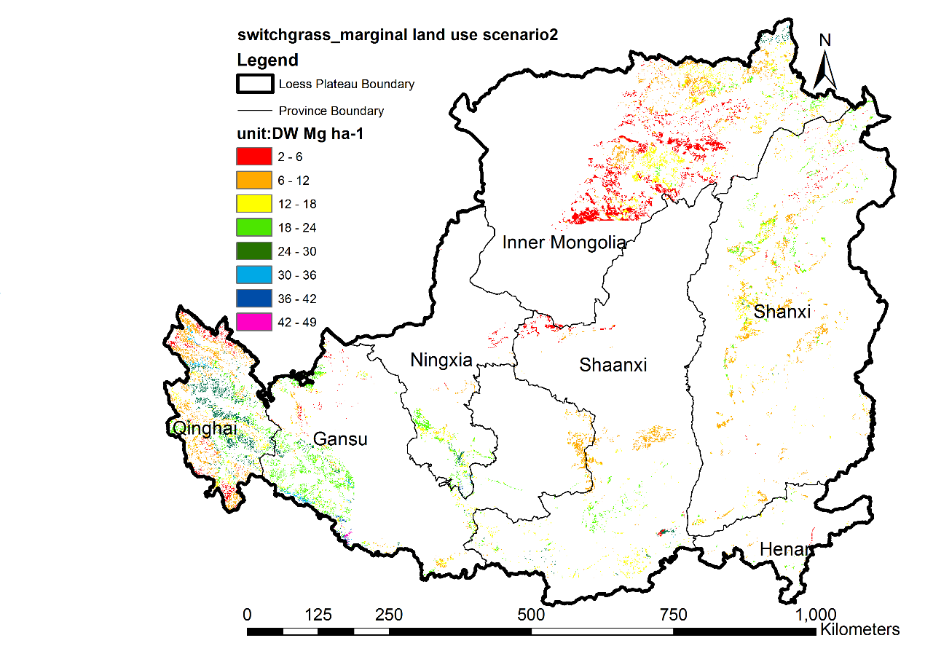
**

**Figure S8. The yield of the switchgrass on the marginal land of the Loess Plateau estimated from the Fuzzy logical model. (a) The switchgrass yield predicted by Fuzzy logical model; (b) Land use scenario1; (c) Land use scenario2.**

**Reference:**

Aurangzaib, Muhammad et al. 2018. “Developmental Morphology and Biomass Yield of Upland and Lowland Switchgrass Ecotypes Grown in Iowa.” *Agronomy* 8(5): 6–8.

Van Esbroeck, G. A., M. A. Hussey, and M. A. Sanderson. 1997. “Leaf Appearance Rate and Final Leaf Number of Switchgrass Cultivars.” *Crop Science* 37(3): 864–70.

Gao, Z. J. et al. 2015. “Diurnal and Seasonal Variations in Photosynthetic Characteristics of Switchgrass in Semiarid Region on the Loess Plateau of China.” *Photosynthetica* 53(4): 489–98.

GAO, Zhi juan et al. 2017. “Photosynthetic Performance of Switchgrass and Its Relation to Field Productivity: A Three-Year Experimental Appraisal in Semiarid Loess Plateau.” *Journal of Integrative Agriculture* 16(6): 1227–35.

Heaton, Emily A., Frank G. Dohleman, and Stephen P. Long. 2008. “Meeting US Biofuel Goals with Less Land: The Potential of Miscanthus.” *Global Change Biology* 14(9): 2000–2014.

Kiniry, J. R., C. R. Tischler, and G. A. Van Esbroeck. 1999. “Radiation Use Efficiency and Leaf CO2 Exchange for Diverse C4 Grasses.” *Biomass and Bioenergy* 17(2): 95–112.

Kiniry, James Robert et al. 2011. “Clash of the Titans: Comparing Productivity Via Radiation Use Efficiency for Two Grass Giants of the Biofuel Field.” *Bioenergy Research* 5(1): 41–48.

Madakadze, I. C. et al. 1998. “Light Interception, Use-Efficiency and Energy Yield of Switchgrass (Panicum Virgatum L.) Grown in a Short Season Area.” *Biomass and Bioenergy* 15(6): 475–82.

Sanderson, M. A., and D. D. Wolf. 1995. “Morphological Development of Switchgrass in Diverse Environments.” *Agronomy Journal* 87(5): 908–15.
